# Machine and Deep Learning for Tuberculosis Detection on Chest X-Rays: Systematic Literature Review

**DOI:** 10.2196/43154

**Published:** 2023-07-03

**Authors:** Seng Hansun, Ahmadreza Argha, Siaw-Teng Liaw, Branko G Celler, Guy B Marks

**Affiliations:** 1 South West Sydney (SWS), School of Clinical Medicine University of New South Wales Sydney Australia; 2 Woolcock Vietnam Research Group Woolcock Institute of Medical Research Sydney Australia; 3 Graduate School of Biomedical Engineering University of New South Wales Sydney Australia; 4 Tyree Institute of Health Engineering (IHealthE) University of New South Wales Sydney Australia; 5 Ageing Future Institute (AFI) University of New South Wales Sydney Australia; 6 WHO Collaborating Centre (eHealth), School of Population Health University of New South Wales Sydney Australia; 7 Biomedical Systems Research Laboratory, School of Electrical Engineering and Telecommunications University of New South Wales Sydney Australia

**Keywords:** chest x-rays, convolutional neural networks, diagnostic test accuracy, machine and deep learning, PRISMA guidelines, risk of bias, QUADAS-2, sensitivity and specificity, systematic literature review, tuberculosis detection

## Abstract

**Background:**

Tuberculosis (TB) was the leading infectious cause of mortality globally prior to COVID-19 and chest radiography has an important role in the detection, and subsequent diagnosis, of patients with this disease. The conventional experts reading has substantial within- and between-observer variability, indicating poor reliability of human readers. Substantial efforts have been made in utilizing various artificial intelligence–based algorithms to address the limitations of human reading of chest radiographs for diagnosing TB.

**Objective:**

This systematic literature review (SLR) aims to assess the performance of machine learning (ML) and deep learning (DL) in the detection of TB using chest radiography (chest x-ray [CXR]).

**Methods:**

In conducting and reporting the SLR, we followed the PRISMA (Preferred Reporting Items for Systematic Reviews and Meta-Analyses) guidelines. A total of 309 records were identified from Scopus, PubMed, and IEEE (Institute of Electrical and Electronics Engineers) databases. We independently screened, reviewed, and assessed all available records and included 47 studies that met the inclusion criteria in this SLR. We also performed the risk of bias assessment using Quality Assessment of Diagnostic Accuracy Studies version 2 (QUADAS-2) and meta-analysis of 10 included studies that provided confusion matrix results.

**Results:**

Various CXR data sets have been used in the included studies, with 2 of the most popular ones being Montgomery County (n=29) and Shenzhen (n=36) data sets. DL (n=34) was more commonly used than ML (n=7) in the included studies. Most studies used human radiologist’s report as the reference standard. Support vector machine (n=5), k-nearest neighbors (n=3), and random forest (n=2) were the most popular ML approaches. Meanwhile, convolutional neural networks were the most commonly used DL techniques, with the 4 most popular applications being ResNet-50 (n=11), VGG-16 (n=8), VGG-19 (n=7), and AlexNet (n=6). Four performance metrics were popularly used, namely, accuracy (n=35), area under the curve (AUC; n=34), sensitivity (n=27), and specificity (n=23). In terms of the performance results, ML showed higher accuracy (mean ~93.71%) and sensitivity (mean ~92.55%), while on average DL models achieved better AUC (mean ~92.12%) and specificity (mean ~91.54%). Based on data from 10 studies that provided confusion matrix results, we estimated the pooled sensitivity and specificity of ML and DL methods to be 0.9857 (95% CI 0.9477-1.00) and 0.9805 (95% CI 0.9255-1.00), respectively. From the risk of bias assessment, 17 studies were regarded as having unclear risks for the reference standard aspect and 6 studies were regarded as having unclear risks for the flow and timing aspect. Only 2 included studies had built applications based on the proposed solutions.

**Conclusions:**

Findings from this SLR confirm the high potential of both ML and DL for TB detection using CXR. Future studies need to pay a close attention on 2 aspects of risk of bias, namely, the reference standard and the flow and timing aspects.

**Trial Registration:**

PROSPERO CRD42021277155; https://www.crd.york.ac.uk/prospero/display_record.php?RecordID=277155

## Introduction

Prior to the COVID-19 pandemic, tuberculosis (TB) was the leading infectious cause of mortality globally [1-3]. Many people with TB do not have symptoms and, therefore, chest radiography has an important role in the detection, and subsequent diagnosis, of patients with this disease [4,5]. Traditionally, chest radiographs have required expert clinicians (usually radiologists or chest physicians) to interpret radiographic images, but this method is expensive and, furthermore, there is substantial within- and between-observer variability, indicating poor reliability of human readers [6]. Therefore, there has been substantial work in utilizing various artificial intelligence (AI)–based algorithms to address the limitations of human reading of chest radiographs for diagnosing TB.

Considered as the most important subdomain in AI, machine learning (ML) has gained increasing popularity in the last 2 decades, although it has been in use since the introduction of artificial neural network many years earlier [7]. It can be seen as a set of methods that can learn from input data, build a model, and improve its analyses to make informed decisions [8,9]. Various ML algorithms have been developed and applied to tackle many problems in many different fields, including TB-related research [10].

Liu et al [11], for example, developed a neural network (NN) system to diagnose TB disease using chest radiographs. Using the proposed system, they could obtain quite high accuracy scores from 89.0% to 96.1% on 3 different data sets. Similarly, Khan et al [12] proposed an NN for the classification task of differentiating between positive TB and negative TB classes on more than 12,600 patient records. The overall accuracy of the proposed NN model was more than 94%. Ghanshala et al [13] compared various ML techniques, including support vector machine (SVM), k-nearest neighbor (kNN), random forest (RF), and NN for effective identification of TB. From their experimental results, they found that the NN classifier performed better than other classifiers to detect TB with an accuracy of 80.45%. Lastly, Chandra et al [14] recently proposed an automatic technique to detect abnormal chest x-ray (CXR) images with 1 or more pathologies, such as pleural effusion, infiltration, or fibrosis due to TB disease. They used SVM with hierarchical feature extraction and found promising results with accuracy ranging from 95.6% to 99.4% on 2 public data sets used in the study.

As a new form of AI, or more specifically ML, deep learning (DL) has gained traction recently due to the availability of increasing computation power and abundant data volume. DL originated from the NN concept that uses more hierarchical layers to segregate and manage the final output [8]. It requires less time-consuming preprocessing and feature engineering than other traditional methods (including ML ones) and is more accurate [15,16]. DL methods have been widely used in TB-related studies.

Lakhani and Sundaram [17] conducted a study to evaluate the efficacy of deep convolutional neural networks (CNNs) for detecting TB on chest radiographs. They used an ensemble of AlexNet and GoogLeNet and achieved 98.7% accuracy. Similarly, Hooda et al [18] presented an ensemble DL-based TB detection system based on AlexNet, GoogLeNet, and ResNet and 4 different data sets. The ensemble method could attain an accuracy score of 88.24%. In another work, Heo et al [19] used various DL approaches to detect TB in chest radiographs of annual workers’ health examination data. Five CNN architectures, including VGG-19, InceptionV3, ResNet-50, DenseNet-121, and InceptionResNetV2, have been employed on CXR, while 1 CNN model (VGG-19) was combined with demographic variables (age, weight, height, and gender). They found that a model using a combination of CXR and demographic data could perform better than the models using CXR alone. Lastly, Sathitratanacheewin et al [20] developed a deep CNN model based on InceptionV3 for automated classification of TB-related CXR. The experimental results on 2 data sets gave area under the curve (AUC) scores ranging from 0.7054 to 0.8502.

In our previous review of the literature with a focus on AI-based TB detection, from 33 included studies, most (n=20) used radiographic biomarkers rather than physiological and molecular biomarkers. Moreover, most of the included studies used DL approaches (n=21) rather than ML approaches, with the most applied DL architectures being AlexNet, ResNet-50, VGG-16, and GoogLeNet. One interesting finding is that ML approaches have better overall accuracy and specificity than the DL. By contrast, the DL approaches have better AUC and sensitivity than the ML approaches. This might be rooted in the data volume and quality available when implementing the former. Furthermore, from the systematic review, we also found that AI-based algorithms have moderate to high specificity and sensitivity for TB detection. This confirms the potential value of AI-based algorithms for TB detection. However, very few studies have focused on implementing AI-based algorithms for early detection of TB, and this warrants further study.

In this review, we aim to evaluate the performance of available ML and DL algorithms developed to detect TB from CXR data. This was motivated by findings from previous reviews that many related studies used radiographic biomarkers, especially in the form of CXR images. However, in contrast to our previous review that took a broader focus on AI methods for TB detection and early TB detection, in this review, we put more focus on ML and DL efficacies for TB detection using CXR.

There are several other review articles in this domain. Singh et al [21] performed a narrative review that focused on the limitations of conventional TB diagnostics and broad applications of ML and DL in TB diagnosis. They also summarized several established industrial-grade tools, such as CAD4TB, Lunit INSIGHT, qXR, and InferRead DR Chest, as prospective AI-assisted tools in TB diagnosis. This differed from our current review where we performed a systematic literature review (SLR) to assess the performance results of ML and DL in TB detection using a specific form of data set, the CXR. Harris et al [22] conducted a systematic review focusing on the diagnostic accuracy of AI-based software for the identification of pulmonary TB (PTB) on CXR. This is similar to the focus of our review. However, in their study, the main comparison was conducted for 2 computer-aided detection design methods, namely, ‘development’ and ‘clinical’ studies. In our review, the main comparison is on the efficacies of ML versus DL methods. We only consider studies that have clearly defined the ML or DL methods used for TB detection using CXR. Lastly, Santosh et al [23] also conducted a systematic review with a very specific focus on DL for TB screening using CXR. They reviewed 54 records that had been published between 2016 and 2021. This differed from our review where we also included ML methods as a key element to be compared. We also performed the SLR with a wider time frame not limited to a specific period to obtain a better understanding of the trend of ML and DL applications in TB detection using CXR.

In the following section, we describe the methods applied in conducting this SLR. It consists of the following subsections: Information Sources, Search Strategy, Inclusion and Exclusion Criteria, Data Extracted, Outcomes Assessed, Strategy for Data Analysis and Synthesis, Potential for Publication Bias. Next, in the “Results” section, we describe the General Characteristics of the Included Studies, Risk of Bias Assessment Result, and the review Study Results. In the “Discussion” section, first we explain the Principal Findings, followed by Limitations and Conclusions from this SLR.

## Methods

### Design

In conducting this SLR, we followed the PRISMA (Preferred Reporting Items for Systematic Reviews and Meta-Analyses) guidelines [24]. We started by preparing the review protocol based on the PRISMA-Protocol 2015 Statement [25,26] and registered the protocol on PROSPERO (Prospective Register of Systematic Reviews), the world’s first international database of prospectively registered systematic reviews launched in February 2011 to increase the transparency of SLRs [27]. The protocol is available at PROSPERO with Record ID CRD42021277155 [28]. As this SLR focuses on retrospective studies, no ethical approval was required.

### Information Sources

In this SLR, we collected the records from 3 major databases, namely, Scopus, PubMed, and IEEE (Institute of Electrical and Electronics Engineers). Those are recommended academic search systems for systematic reviews and meta-analyses [29]. We checked for all available literature in each database up to May 9, 2021, when this SLR was started.

### Search Strategy

There are several main keywords used for the search strategy, including “Artificial Intelligence,” “Tuberculosis,” “Detection,” and “Chest Radiograph*.” Moreover, several alternative synonyms for each keyword are included for the searching process in those databases. Textbox 1 shows the main keywords together with their alternative synonyms that were proposed by SH and refined by AA.

Using the keywords and alternative terms, we obtained results as shown in Table 1. There were a total of 328 records, but only 309 records were available for download. The list of not downloaded records is presented in Multimedia Appendix 1.

**Main keywords (italics) and alternative terms for the search strategy.**

- *Artificial intelligence* (AI, deep learning, DL, machine learning, ML, predictive analysis)
- *Tuberculosis* (TB, pulmonary tuberculosis [PTB])
- *Detection* (detect*, diagnosis)
- Chest radiograph* (chest x-ray*, CXR, radiograph image*)

The * represents the wild-type character that can be used to represent any available characters in the search engine.

**Table 1:** Searched keywords and results.

Keywords	Scopus	PubMed	IEEE^a^
(TITLE-ABS-KEY ("Deep Learning" OR "DL" OR "Machine Learning" OR "ML" OR "Artificial Intelligence" OR "AI" OR "Predictive Analytics") AND TITLE-ABS-KEY (tuberculosis OR "TB" OR "Pulmonary Tuberculosis" OR "PTB") AND TITLE-ABS-KEY (detection OR detect* OR diagnosis) AND TITLE-ABS-KEY ("Chest Radiograph*" OR "Chest X-ray*" OR "CXR" OR "Radiograph Image*") ) AND (LIMIT-TO (DOCTYPE , "ar") OR LIMIT-TO (DOCTYPE , "cp")) AND (LIMIT-TO ( LANGUAGE, "English"))	196	87	45
Available for download (N=309)	185	79	45

^a^Institute of Electrical and Electronics Engineers.

### Inclusion and Exclusion Criteria

Textbox 2 shows the inclusion and exclusion criteria adopted in this SLR. From 309 downloaded records, we screened all records based on the inclusion and exclusion criteria determined.

**Inclusion and exclusion criteria.**

**Inclusion criteria**

- Full-text articles in peer-reviewed journals or proceedings
- Written in English
- Focused on tuberculosis (TB) or pulmonary TB, chest radiograph, or chest x-ray
- Applied machine learning or deep learning algorithms

**Exclusion criteria**

- Literature review, case reports, letters, corrigendum, editorial commentary
- Not published in English
- Focused on extrapulmonary TB, latent TB, and other types of data
- Applied statistical methods, nonartificial intelligence methods, and general artificial intelligence methods not considered as machine learning or deep learning

### Data Extracted

Information extracted from each included study included the following: (1) title, (2) authors, (3) published year, (4) journal or proceeding’s title, (5) study objectives, (6) study findings, (7) data set characteristics and size, (8) parameters (metrics) used, (9) ML and DL methods applied, (10) best performance results, (11) comparison with other studies, (12) outcome types, (13) funding or sponsor sources, and (14) Google citation counts.

### Outcomes Assessed

The main outcome of this SLR is the list of various ML and DL methods for TB detection based on chest radiograph images. The secondary outcome is the summary statistics of diagnostic performance of various ML and DL methods for TB detection on chest radiograph, including accuracy, AUC, sensitivity, and specificity.

### Strategy for Data Analysis and Synthesis

A narrative synthesis was presented in the text based on extracted information from included studies. Descriptive statistics was performed mainly using box and whisker plots and other tables or figures to summarize and describe the characteristics and key findings of the included studies. The narrative synthesis was used to explore the relationship and findings both within and between the included studies.

For the quantitative analysis, we performed the meta-analysis for diagnostic test accuracy. This particular meta-analysis differs from the meta-analysis of therapeutic or interventional studies as it is necessitated analyzing simultaneously a pair of outcome measures (sensitivity and specificity) instead of a single outcome [30]. In this SLR, a simple meta-analysis for diagnostic test accuracy was conducted on included studies that provide confusion matrix results.

### Potential for Publication Bias

To assess the risk of bias within the included studies, we used a modified Quality Assessment of Diagnostic Accuracy Studies version 2 (QUADAS-2) tool, which is recommended for evaluating the risk of bias and applicability of primary diagnostic accuracy studies in SLRs [31]. There are 4 key domains in QUADAS-2: (1) patient selection, (2) index test, (3) reference standard, and (4) flow and timing. SH and AA performed the assessment independently. In case of disagreements, it was resolved first by asking a third opinion from GBM, and then by discussion and majority voting with all authors if needed.

## Results

### Overview

**Figure 1:**
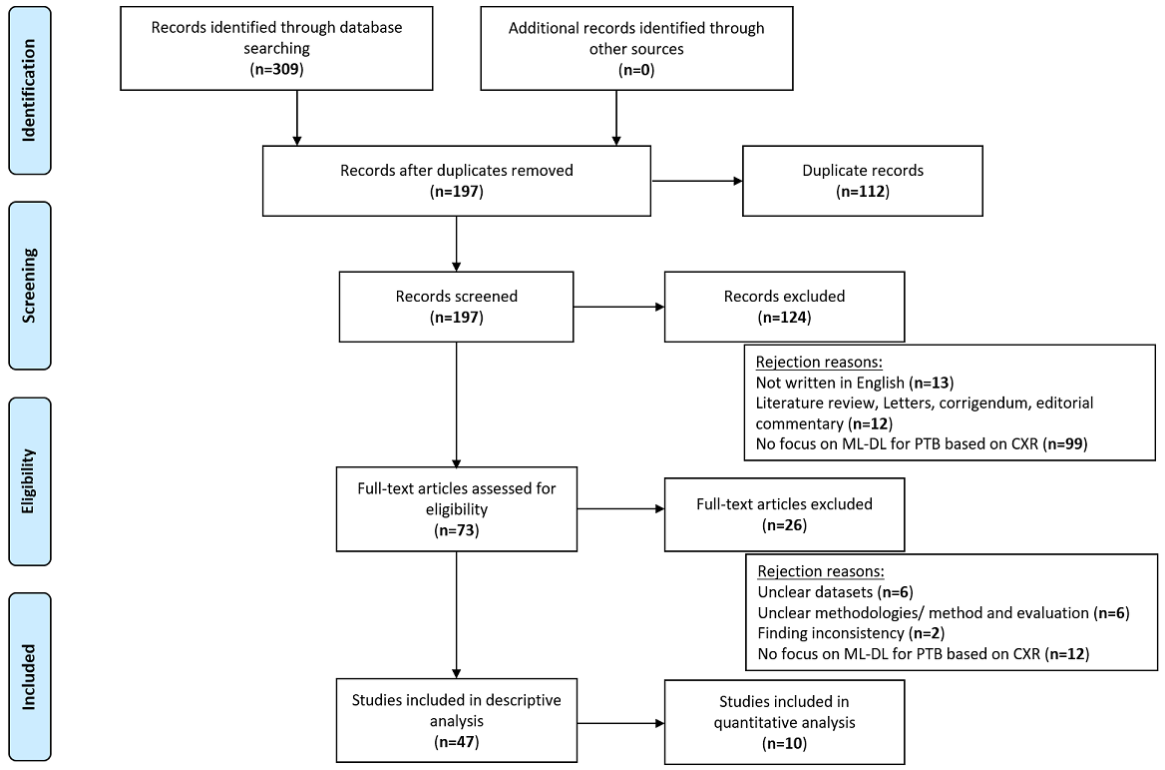
PRISMA compliant SLR. CXR: chest x-ray; DL: deep learning; ML: machine learning; PRISMA: Preferred Reporting Items for Systematic Reviews and Meta-Analyses; PTB: pulmonary tuberculosis; SLR: systematic literature review.

Figure 1 illustrates all the phases conducted throughout the SLR, starting from identification, screening, eligibility, to inclusion of selected studies.

First, 309 records were identified from 3 databases used in this SLR in the identification phase. After removing duplicates (n=112), 197 records remained and these were passed to the screening phase. The records’ titles, abstract, and keywords were examined in this phase using 3 main rejection reasons, namely, (1) not written in English (n=13); (2) being in the form of literature review, case reports, letters, corrigendum, or editorial commentary (n=12); and (3) not focused on ML or DL for detection of TB or PTB based on CXR (n=99). After these exclusions 73 records remained.

Next, in the eligibility phase, the 73 remaining records were assessed by reading their full-text content. A total of 26 records were excluded for the following reasons: (1) has unclear data sets (n=6), (2) has unclear methodologies or method and evaluation (n=6), (3) has finding inconsistency (n=2), and (4) did not focus on ML or DL for TB or PTB detection based on CXR (n=12). After this phase, 47 records were passed to the included phase, which were checked for quality and data extraction. Of the 47 records, 10 were included in the quantitative analysis using data extracted from the confusion matrix results provided in the sources.

### General Characteristics of the Included Studies

Table 2 shows the general characteristics of 47 studies finally selected for this SLR. A total of 41 different CXR sources were used in these studies, including 374,129 images. The 3 most used CXR sources were Shenzhen (SZ; 36/47 studies), Montgomery County (MC; n=29), and ChestX-ray14 or NIH-14 (n=4). SZ and MC are publicly available CXR data sets for computer-aided screening of PTB disease [32], while ChestX-ray14, an extension of ChestX-ray8, is another publicly available data set of 14 common thorax diseases [33].

**Table 2:** General characteristics of included studies.

Study	Data set	Reference standard	Machine learning-deep learning	Best result
Mizan et al [34]	Shenzhen, Montgomery County	Radiologist’s reading	CNNs^a^: DenseNet-169, MobileNet, Xception, and Inception-V3	DenseNet-169 (precision 92%, recall 92%, *F*_1_-score 92%, validation accuracy 91.67%, and AUC^b^ 0.915)
Hwang et al [35]	Korean Institute of Tuberculosis, Montgomery County, Shenzhen	Unclear (Korean Institute of Tuberculosis); radiologist’s reading	Customized CNN based on AlexNet + transfer learning	Customized CNN (AUC 96.7% [Shenzhen] and accuracy 90.5% [Montgomery County])
Hooda et al [36]	Montgomery County, Shenzhen, Belarus, Japanese Society of Radiological Technology	Unclear (Belarus); radiologist’s reading	Proposed (blocks), AlexNet, ResNet, Ensemble (proposed + AlexNet + ResNet)	Ensemble (accuracy 90.0%, AUC 0.96, sensitivity 88.42%, and specificity 92.0%)
Melendez et al [37]	Zambia, Tanzania, Gambia	Radiologist’s reading	kNN^c^, multiple-instance learning–based system: miSVM^d^, miSVM + probability estimation and data discarding, single iteration-maximum pattern margin support vector machine + probability estimation and data discarding	Single iteration-maximum pattern margin support vector machine + probability estimation and data discarding (0.86 [Zambia], 0.86 [Tanzania], and 0.91 [Gambia])
Rajaraman et al [38]	Shenzhen, Montgomery County, Kenya, India	Radiologist’s reading	SVM with GIST, histogram of oriented gradients, speeded up robust features (feature engineering); SVM with AlexNet, VGG-16, GoogLeNet, ResNet-50; and ensemble approach	Ensemble (Shenzhen [accuracy 93.4%, AUC 0.991], Montgomery County [accuracy 87.5%, AUC 0.962], Kenya [accuracy 77.6%, AUC 0.826], and India [accuracy 96.0%, AUC 0.965])
Zhang et al [39]	Jilin, Guangzhou, Shanghai	Unclear	Proposed: Feed-forward CNN model with integrated convolutional block attention module and 4 other CNNs (AlexNet, GoogLeNet, DenseNet, and ResNet-50)	Proposed network (recall/sensitivity 89.7%, specificity 85.9%, accuracy 87.7%, and AUC 0.943)
Melendez et al [40]	Cape Town	Culture	Feature engineering: minimum redundancy maximum relevance—multiple learner fusion: RF^e^ and extremely randomized trees	Multiple learner fusion: RF and extremely randomized trees (AUC 0.84, sensitivity 95%, specificity 49%, and negative predictive value 98%)
Ghanshala et al [13]	Montgomery County, Shenzhen, Japanese Society of Radiological Technology	Radiologist’s reading	SVM, RF, kNN, neural network	Neural network (AUC 0.894, accuracy 81.1%, *F*_1_-score 81.1%, precision 81.1%, recall 81.1%, and average accuracy 80.45%)
Ahsan et al [41]	Montgomery County, Shenzhen	Radiologist’s reading	CNN: VGG-16	VGG-16 + data augmentation (AUC 0.94 and accuracy 81.25%)
Sharma et al [42]	Custom data set	Unclear	A total of 29 different custom artificial intelligence models	Custom deep artificial intelligence model (100% normal, 100% COVID-19, 66.67% new COVID-19, 100% non–COVID-19, 93.75% pneumonia, 80% tuberculosis)
Hooda et al [18]	Montgomery County, Shenzhen, Belarus, Japanese Society of Radiological Technology	Unclear (Belarus); radiologist’s reading	Ensemble of AlexNet, GoogLeNet, and ResNet	Ensemble (accuracy 88.24%, AUC 0.93, sensitivity 88.42%, and specificity 88%)
van Ginneken et al [43]	Netherlands, Interstitial Disease database	Radiologist’s reading	Active shape model segmentation, kNN classifier, weighted multiplier	Proposed scheme with kNN (sensitivity 86%, specificity 50%, and AUC 0.82)
Chandra et al [14]	Montgomery County, Shenzhen	Radiologist’s reading	SVM with hierarchical feature extraction	SVM with hierarchical feature extraction (Montgomery County [accuracy 95.6%, AUC 0.95] and Shenzhen [accuracy 99.4% and AUC 0.99])
Karnkawinpong and Limpiyakorn [44]	Montgomery County, Shenzhen, Thailand	Radiologist’s reading	AlexNet, VGG-16, and CapsNet	CapsNet (accuracy 80.06%, sensitivity 92.72%, and specificity 69.44%)
Stirenko et al [45]	Shenzhen	Radiologist’s reading	Customized CNN	Customized CNN (64% [lossy data augmentation] and 70% [lossless data augmentation])
Rajpurkar et al [46]	Africa	Culture	Customized CNN based on DenseNet-121	CheXaid (accuracy 79%, sensitivity 67%, and specificity 87%)
Sivaramakrishnan et al [47]	Shenzhen, Montgomery County, Kenya, India	Radiologist’s reading	Customized CNN, AlexNet, VGG-16, VGG-19, Xception, and ResNet-50	Proposed pretrained CNNs (accuracy 85.5% [Shenzhen], 75.8% [Montgomery County], 69.5% [Kenya], and 87.6% [India]; AUC 0.926 [Shenzhen], 0.833 [Montgomery County], 0.775 [Kenya], and 0.956 [India])
Owais et al [48]	Shenzhen, Montgomery County	Radiologist’s reading	Ensemble-shallow–deep CNN + multilevel similarity measure algorithm	Ensemble on Montgomery County (*F*_1_-score 0.929, average precision 0.937, average recall 0.921, accuracy 92.8%, and AUC 0.965)
Xie et al [49]	Japanese Society of Radiological Technology, Shenzhen, Montgomery County, local from the First Affiliated Hospital of Xi’an Jiao Tong University	Radiologist’s reading	Segmentation: U-Net; classification: proposed method based on Faster region-based convolutional network + feature pyramid network	Faster region-based convolutional network + feature pyramid network (Shenzhen [AUC 0.941, accuracy 90.2%, sensitivity 85.4%, and specificity 95.1%], Montgomery County [AUC 0.977, accuracy 92.6%, sensitivity 93.1%, and specificity 92.3%], Local First Affiliated Hospital of Xi’an Jiao Tong University [AUC 0.993, accuracy 97.4%, sensitivity 98.3%, and specificity 96.2%])
Andika et al [50]	Shenzhen	Radiologist’s reading	Customized CNN	Customized CNN: normal (precision 83% and recall 83%); pulmonary tuberculosis (precision 84% and recall 84%); overall accuracy 84%
Das et al [51]	Shenzhen, Montgomery County	Radiologist’s reading	InceptionNet V3 and modified (truncated) InceptionNet V3	Modified InceptionNet V3: Shenzhen train Montgomery County test (accuracy 76.05%, AUC 0.84, sensitivity 63%, specificity 81%, and precision 89%); Montgomery County train Shenzhen test (accuracy 71.47%, AUC 0.79, sensitivity 59%, specificity 73%, and precision 84%); and combined (accuracy 89.96%, AUC 0.95, sensitivity 87%, specificity 93%, and precision 92%)
Gozes and Greenspan [52]	ChestX-ray14, Montgomery County, Shenzhen	Radiologist’s reading	MetaChexNet based on DenseNet-121	MetaChexNet: Shenzhen AUC 0.965, Montgomery County AUC 0.928, and combined AUC 0.937
Hooda et al [53]	Shenzhen, Montgomery County	Radiologist’s reading	Proposed CNN	Proposed CNN: accuracy 82.09% and loss 0.4013
Heo et al [19]	Yonsei	Radiologist’s reading	VGG19, InceptionV3, ResNet50, DenseNet121, InceptionResNetV2, and CNN with demographic variables (VGG19 + demographic variables)	CNN with demographic variables (VGG19 AUC 0.9213) and CNN with image-only information (VGG19 0.9075)
Lakhani and Sundaram [17]	Shenzhen, Montgomery County, Belarus, Thomas Jefferson University Hospital	Culture (Belarus and Thomas Jefferson); radiologist’s reading (all data sets)	Ensemble of AlexNet and GoogLeNet	Ensemble (AUC 0.99); Ensemble + radiologist augmented (sensitivity 97.3%, specificity 100%, and accuracy 98.7%)
Sathitratanacheewin et al [20]	Shenzhen, ChestX-ray8	Radiologist’s reading	Proposed CNN based on Inception V3	Proposed CNN (Shenzhen AUC 0.8502) and ChestX-ray8 (AUC 0.7054)
Dasanayaka and Dissanayake [54]	Shenzhen, Montgomery County, Medical Information Mart for Intensive Care, and Synthesis	Unclear (Medical Information Mart for Intensive Care and Synthesis); radiologist’s reading	Proposed CNN based on generative adversarial network, UNET, and ensemble of VGG16 + InceptionV3	Ensemble (Youden’s index 0.941, sensitivity 97.9%, specificity 96.2%, and accuracy 97.1%)
Nguyen et al [55]	Shenzhen, Montgomery County, National Institutes of Health-14	Radiologist’s reading	ResNet-50, VGG16, VGG19, DenseNet-121, and Inception ResNet	DenseNet (Shenzhen AUC 0.99 and Montgomery County AUC 0.80)
Meraj et al [56]	Shenzhen, Montgomery County	Radiologist’s reading	VGG-16, VGG-19, ResNet50, and GoogLeNet	VGG-16: Shenzhen (accuracy 86.74% and AUC 0.92), Montgomery County (accuracy 77.14% and AUC 0.75), and VGG-19 (AUC 0.90)
Becker et al [57]	Uganda	Unclear	ViDi—industrial-grade deep learning image analysis software (suite version 2.0, ViDi Systems)	ViDi software (overall AUC 0.98)
Hwang et al [58]	Seoul National University Hospital, Boramae, Kyunghee, Daejeon Eulji, Montgomery County, Shenzhen	Culture (Seoul National University Hospital, Boramae, Kyunghee, Daejeon); radiologist’s reading	Proposed CNN	Proposed CNN (AUC 0.977-1.000, area under the alternative free-response receiver operating characteristics curves 0.973-1.000, sensitivity 94.3%-100%, specificity 91.1%-100%, and true detection rate 94.5%-100%)
Pasa et al [59]	Montgomery County, Shenzhen, Belarus	Unclear (Belarus); radiologist’s reading	Proposed CNN	Proposed CNN: Montgomery County (accuracy 79.0% and AUC 0.811), Shenzhen (accuracy 84.4% and AUC 0.900), and combined 3 data sets (accuracy 86.2% and AUC 0.925)
Ahmad Hijazi et al [60]	Shenzhen, Montgomery County	Radiologist’s reading	Ensemble of InceptionV3, VGG-16, and a custom-built architecture	Ensemble (accuracy 91.0%, sensitivity 89.6%, and specificity 90.7%)
Hwa et al [61]	Shenzhen, Montgomery County	Radiologist’s reading	Ensemble of InceptionV3 and VGG-16	Ensemble + canny edge (accuracy 89.77%, sensitivity 90.91%, and specificity 88.64%)
Ayaz et al [62]	Shenzhen, Montgomery County	Radiologist’s reading	Ensemble (pretrained CNNs: InceptionV3, InceptionResnetv2, VGG16, VGG19, MobileNet, ResNet50, and Xception) with Gabor filter	Ensemble with Gabor filter: Montgomery County (accuracy 93.47% and AUC 0.97) and Shenzhen (accuracy 97.59% and AUC 0.99)
Govindarajan and Swaminathan [63]	Montgomery County	Radiologist’s reading	ELM^f^ and online sequential ELM	ELM (accuracy 99.2%, sensitivity 99.3%, specificity 99.3%, precision 99.0%, *F*_1_-score 99.2%, and Matthews correlation coefficient 98.6%) and online sequential ELM (accuracy 98.6%, sensitivity 98.7%, specificity 98.7%, precision 97.9%, *F*_1_-score 98.6%, and Matthews correlation coefficient 97.0%)
Rashid et al [64]	Shenzhen	Radiologist’s reading	Ensemble of ResNet-152, Inception-ResNet-v2, and DenseNet-161 + SVM	Ensemble with SVM (accuracy 90.5%, sensitivity 89.4%, specificity 91.9%, and AUC 0.95)
Munadi et al [65]	Shenzhen	Radiologist’s reading	Image enhancements: unsharp masking, high-frequency emphasis filtering, and contrast-limited adaptive histogram equalization—deep learning (ResNet-50, EfficientNet-B4, and ResNet-18)	Proposed EfficientNet-B4 + unsharp masking (accuracy 89.92% and AUC 0.948)
Abbas and Abdelsamea [66]	Montgomery County	Radiologist’s reading	AlexNet	AlexNet (AUC 0.998, sensitivity 99.7%, and specificity 99.9%)
Melendez et al [67]	Zambia	Radiologist’s reading	Multiple-instance learning + active learning	Multiple-instance learning + active learning (pixel-level AUC 0.870)
Khatibi et al [68]	Montgomery County, Shenzhen	Radiologist’s reading	Logistic regression, SVM with linear and radial basis function kernels, decision tree, RF, and AdaBoost—CNNs (VGG-16, VGG-19, ResNet-101, ResNet-150, DenseNet, and Xception)	Proposed stacked ensemble: Montgomery County (accuracy 99.26%, AUC 0.99, sensitivity 99.42%, and specificity 99.15%) and Shenzhen (accuracy 99.22%, AUC 0.98, sensitivity 99.39%, and specificity 99.47%)
Kim et al [69]	ChestX-ray14, Montgomery County, Shenzhen, Johns Hopkins Hospital	Culture (Johns Hopkins Hospital); radiologist’s reading	ResNet-50 and TBNet	TBNet on Johns Hopkins Hospital (AUC 0.87, sensitivity 85%, specificity 76%, positive predictive value 0.64, and negative predictive value 0.9) and Majority VoteTBNet and 2 radiologists (sensitivity 94%, specificity 85%, positive predictive value 0.76, and negative predictive value 0.96)
Rahman et al [70]	Kaggle, National Library of Medicine, Belarus, National Institute of Allergy and Infectious Diseases TB data set, Radiological Society of North America CXR data set	Unclear (Kaggle, Belarus, National Institute of Allergy and Infectious Diseases, Radiological Society of North America); radiologist’s reading	Lung segmentation—U-Net; classification—MobileNetv2, SqueezeNet, ResNet18, Inceptionv3, ResNet 50, ResNet101, CheXNet, VGG19, and DenseNet201	Without segmentation: CheXNet (accuracy 96.47%, precision 96.62%, sensitivity 96.47%, *F*_1_-score 96.47%, and specificity 96.51%); with segmentation: DenseNet201 (accuracy 98.6%, precision 98.57%, sensitivity 98.56%, *F*_1_-score 98.56%, and specificity 98.54%)
Yoo et al [71]	ChestX-ray14, Shenzhen, East Asian Hospital	Unclear (East Asian Hospital); radiologist’s reading	ResNet18	ResNet18: AXIR1 (accuracy 98%, sensitivity 99%, specificity 97%, precision 97%, and AUC 0.98) and AXIR2 (accuracy 80%, sensitivity 72%, specificity 89%, precision 87%, and AUC 0.80)
Oloko-Oba and Viriri [72]	Shenzhen	Radiologist’s reading	Proposed ConvNet	Proposed ConvNet (accuracy 87.8%)
Guo et al [73]	Shenzhen, National Institutes of Health	Radiologist’s reading	Artificial bee colony (VGG16, VGG19, Inception V3, ResNet34, and ResNet50) and ResNet101 (proposed ensemble CNN)	Ensemble: Shenzhen (accuracy 94.59%-98.46%, specificity 95.57%-100%, recall 93.66%-98.67%, *F*_1_-score 94.7%-98.6%, and AUC 0.986-0.999) and National Institutes of Health (accuracy 89.56%-95.49%, specificity 96.69%-98.50%, recall 78.52%-90.91%, *F*_1_-score 85.5%-94.0%, and AUC 0.934-0.976)
Ul Abideen et al [74]	Shenzhen, Montgomery County	Radiologist’s reading	Proposed Bayesian convolutional neural network	Bayesian convolutional neural network: Montgomery County (accuracy 96.42%) and Shenzhen (accuracy 86.46%)

^a^CNN: convolutional neural network.

^b^AUC: area under the curve.

^c^kNN: k-nearest neighbor.

^d^miSVM: multiple instance support vector machine/ maximum pattern margin support vector machine.

^e^RF: random forest.

^f^ELM: extreme learning machine.

Twenty-five of the included studies [14,17-19,34,36-38,47-49,52,53,56,59,62-66,68,70,72-74] reported comparison results with several other previous studies, while the remaining 22 did not. Twenty-six of 47 included studies [19,20,35,38-40,42,43,45,47-50,53,55,56,58-62,65, 69-71,74] were funded or sponsored by companies, private or university research institutions, and governmental institutions, while for the remaining 21 studies no funding sources were identified. Most of the included studies focused on the development of a model or architecture as the proposed solution. Only 2 studies [46,55] developed and built an application for the proposed solution, and another study [57] focused on the diagnostic performance of the commercial software. Almost all included studies were published in the last 5 years, while only 1 study [43] was published in 2002 and considered as one of the early publications that applied AI methods in detecting abnormalities in the chest radiograph. A more detailed analysis of the extracted characteristics is presented as Multimedia Appendix 2.

### Risk of Bias Assessment Result

**Figure 2:**
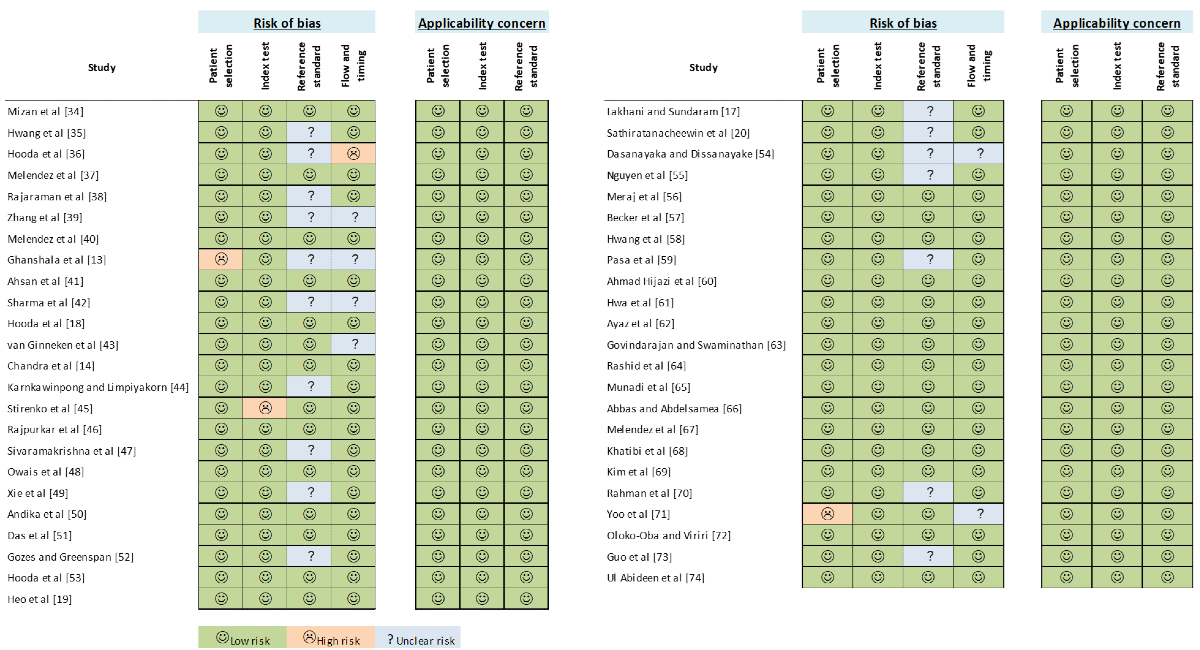
Quality Assessment of Diagnostic Accuracy Studies version 2 (QUADAS-2) assessment results of included studies.

**Figure 3:**
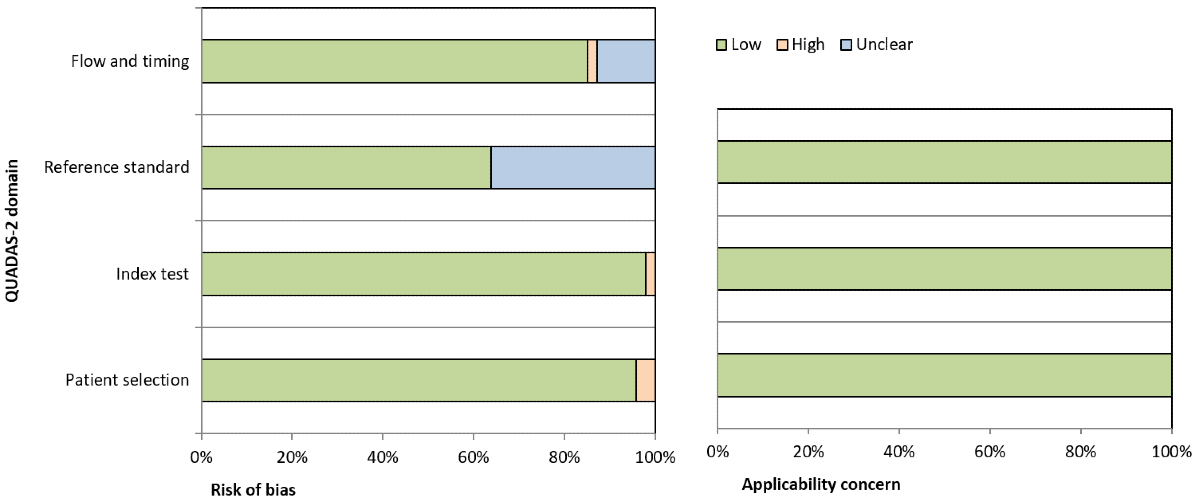
Graphical representation of the Quality Assessment of Diagnostic Accuracy Studies version 2 (QUADAS-2) assessment results of included studies.

Figures 2-3 illustrate the QUADAS-2 assessment results regarding the risk of bias and applicability concerns of included studies. There were 2 studies [13,71] that have a high risk of bias in terms of “patient selection.” This is mainly due to incomplete information on data selection. One study [45] was identified to have a high risk of bias in terms of “index test” due to missing mandatory information of model architecture and hyperparameters being deployed in the study. No high risk of bias in terms of “reference standard” was detected; however, 17 of the included studies [13,17,20,35, 36,38,39,42,44,47,49,52,54,55,59,70,73] did not provide explicit and clear information about reference standards applied for the diagnosis of TB. Hence, we further explored available data sources and publications cited in those included studies to find the reference standards being used. As shown in Table 2, the most commonly used reference standard was a report of a human radiologist. A few studies applied a microbiological reference standard, that is, mycobacterial culture. However, reference standards for several custom and nonpublic data sets could not be determined and are labeled as “unclear” in Table 2.

In terms of “flow and timing,” 6 studies [13,39,42,43,54,71] were categorized as having an unclear risk of bias, and 1 study [36] was assessed with a high risk of bias. These are mainly because no clear information was given regarding the time interval and intervention given between index test(s) and the reference standard used in those studies. Regarding the applicability concerns, all 47 included studies had low concerns, meaning proposed solutions in those studies are feasible and applicable in detecting TB on CXR using ML and DL methods.

### Study Results

Various ML and DL methods have been applied in the included studies: 7/47 (15%) studies [13,14,37,40,43,63,67] focused on using ML approaches, while 34/47 (72%) studies [17-20,34-36,39,41,44-56,58-62,65,66,69-72,74] used DL approaches; 4/47 (9%) studies [38,64,68,73] used both ML and DL approaches, while 2/47 (4%) [42,57] focused on industrial-grade DL image analysis software and various deep AI models without further information on the types of AI techniques used.

**Figure 4:**
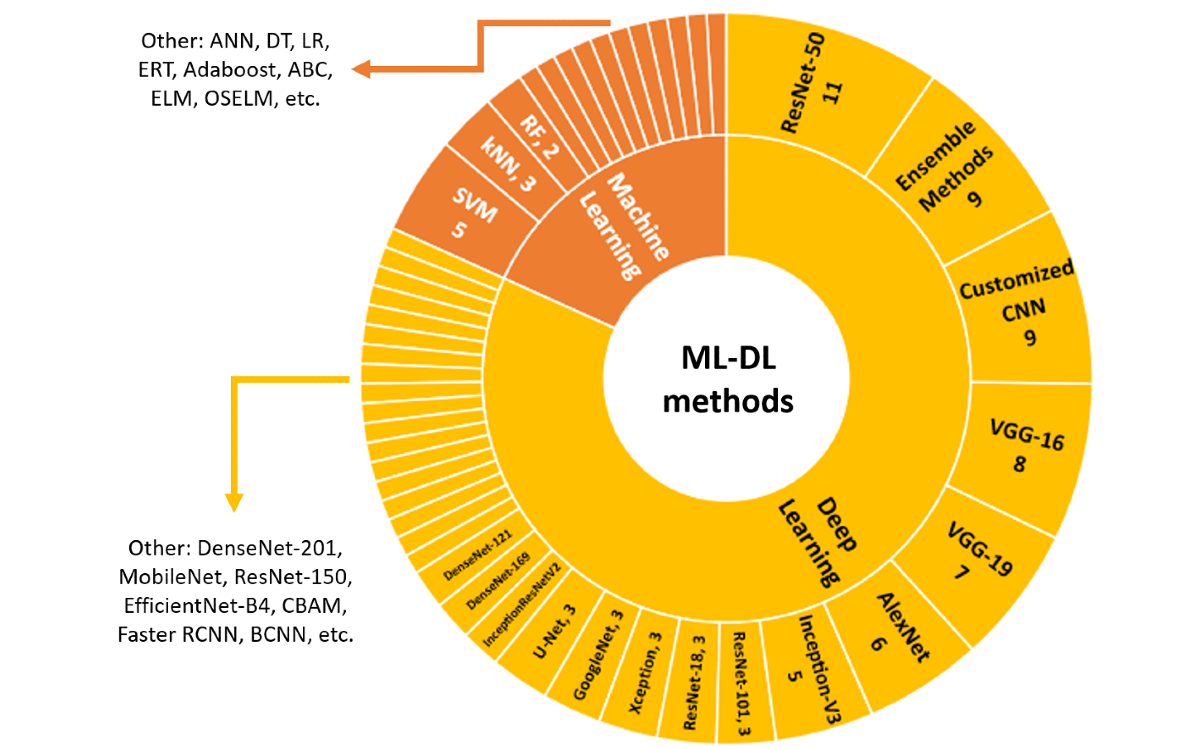
Summary of ML-DL methods employed in included studies. ANN: artificial neural network; CNN: convolutional neural network; DL: deep learning; DT: decision tree; ELM: extreme learning machine; ERT: extremely randomized trees; kNN: k-nearest neighbor; LR: logistic regression; ML: machine learning; OSELM: online sequential extreme learning machine; RF: random forest; SVM: support vector machine.

The most popular DL architectures used in the included studies were ResNet-50 (n=11), followed by VGG-16 (n=8), VGG-19 (n=7), and AlexNet (n=6). However, it is noteworthy that various DL ensemble (n=9) [17,18,36,48,54,60-62,64] and custom (n=9) [20,35,45-47,50,53,58,59] methods were also introduced by authors in this field. For the ML approaches, SVM (n=5) was the most applied method, followed by KNN (n=3) and RF (n=2). Figure 4 depicts the distribution of the ML-DL methods employed in the included studies, noting that more than 1 ML and DL methods might be applied by a study.

**Figure 5:**
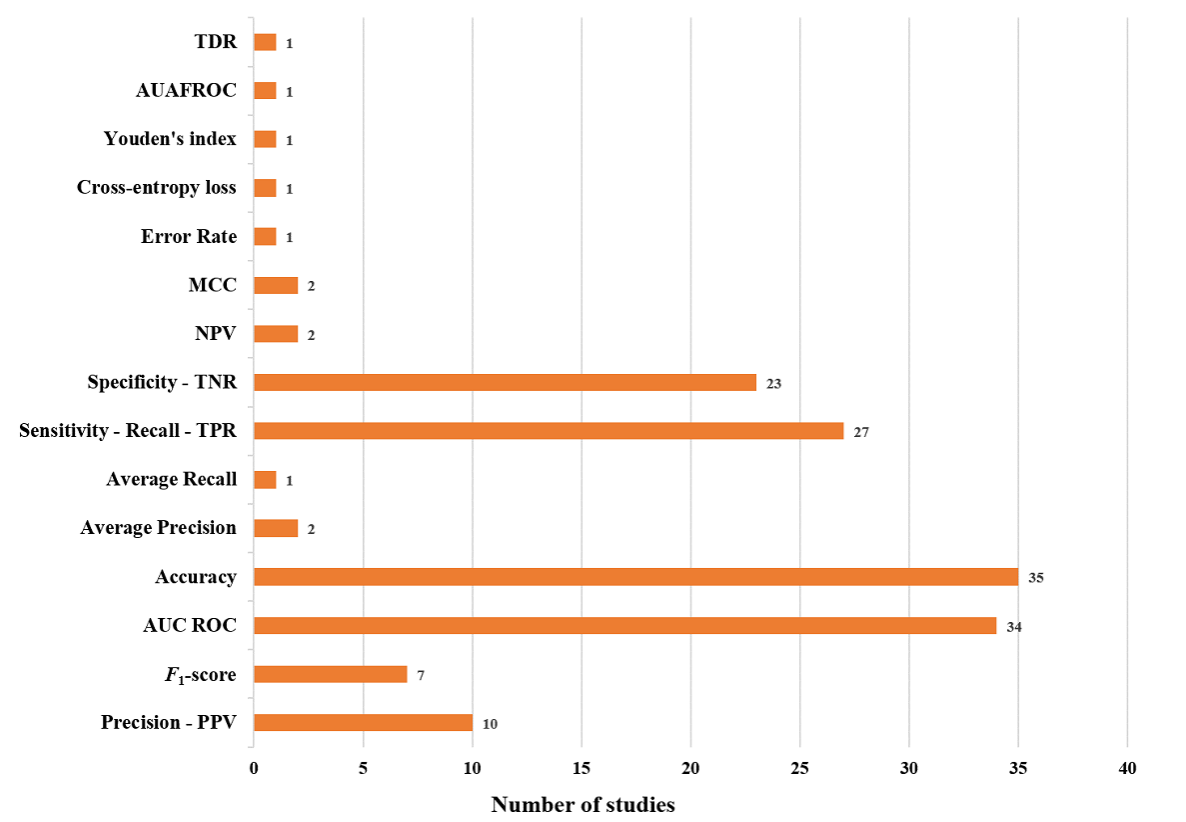
Performance metrics proportion in included studies. AUAFROC: area under the alternative free-response ROC curves; AUC: area under the curve; MCC: Matthews correlation coefficient; NPV: negative predictive value; PPV: positive predictive value; ROC: receiver operating characteristic curve; TDR: true detection rate; TNR: true negative rate; TPR: true positive rate.

In terms of performance measurements being used in the included studies, accuracy (35/47, 74%), AUC (34/47, 72%), sensitivity (27/47, 57%), and specificity (23/47, 49%) were the most used ones. Accuracy is the proportion of all cases that are either true positives or true negatives, while AUC is derived from the receiver operating characteristics curve and is another quantitative measure of model accuracy [75]. Sensitivity and specificity are common metrics in the clinical test domain. Sensitivity is defined as the ability of a model/test to correctly identify people with a condition, while specificity is the ability of a model/test to correctly identify people without a condition [76,77]. Figure 5 shows the proportion of the performance metrics used in the included studies. The full description of performance metrics used in each corresponding study is presented in Multimedia Appendices 2 and 3.

**Figure 6:**
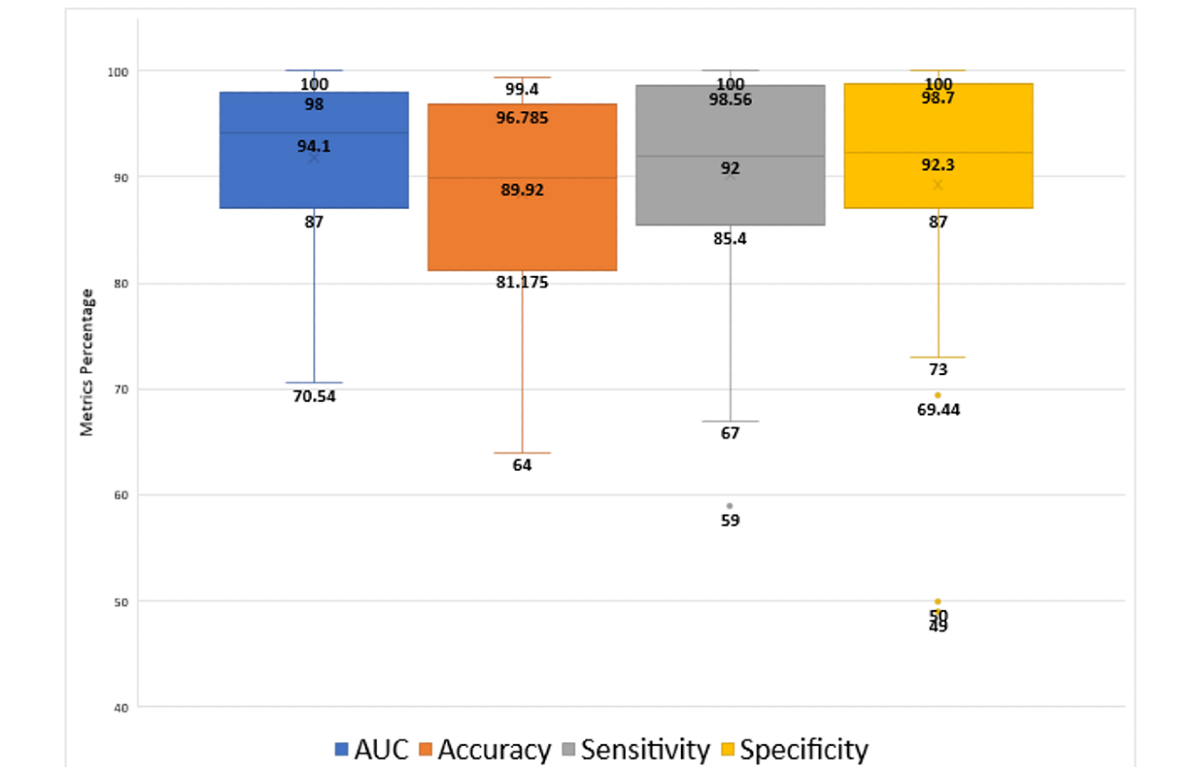
Overall performance of both machine learning (ML) and deep learning (DL)-based methods reviewed. AUC: area under the curve.

The overall performance results of the methods reviewed in this study, in terms of accuracy, AUC, sensitivity, and specificity of all included studies, are shown as a box plot in Figure 6. As seen, accuracy ranged from 64% [45] to 99.4% [14] with a mean value of 88.38% and median value of 89.92%; AUC ranged from 70.54% [20] to 100% [58] with a mean value of 91.78% and median of 94.1%; sensitivity ranged from 59% [51] to 100% [58] with a mean value of 90.15% and median of 92%; and specificity ranged from 49% [40] to 100% [17,58,73] with a mean value of 89.31% and median value of 92.3%. All performance metrics are negatively skewed and have relatively same distribution values. However, there are some outliers detected, 1 for sensitivity at 59% [51] and 3 for specificity at 49% [40], 50% [43], and 69.44% [44] of the included studies.

**Figure 7:**
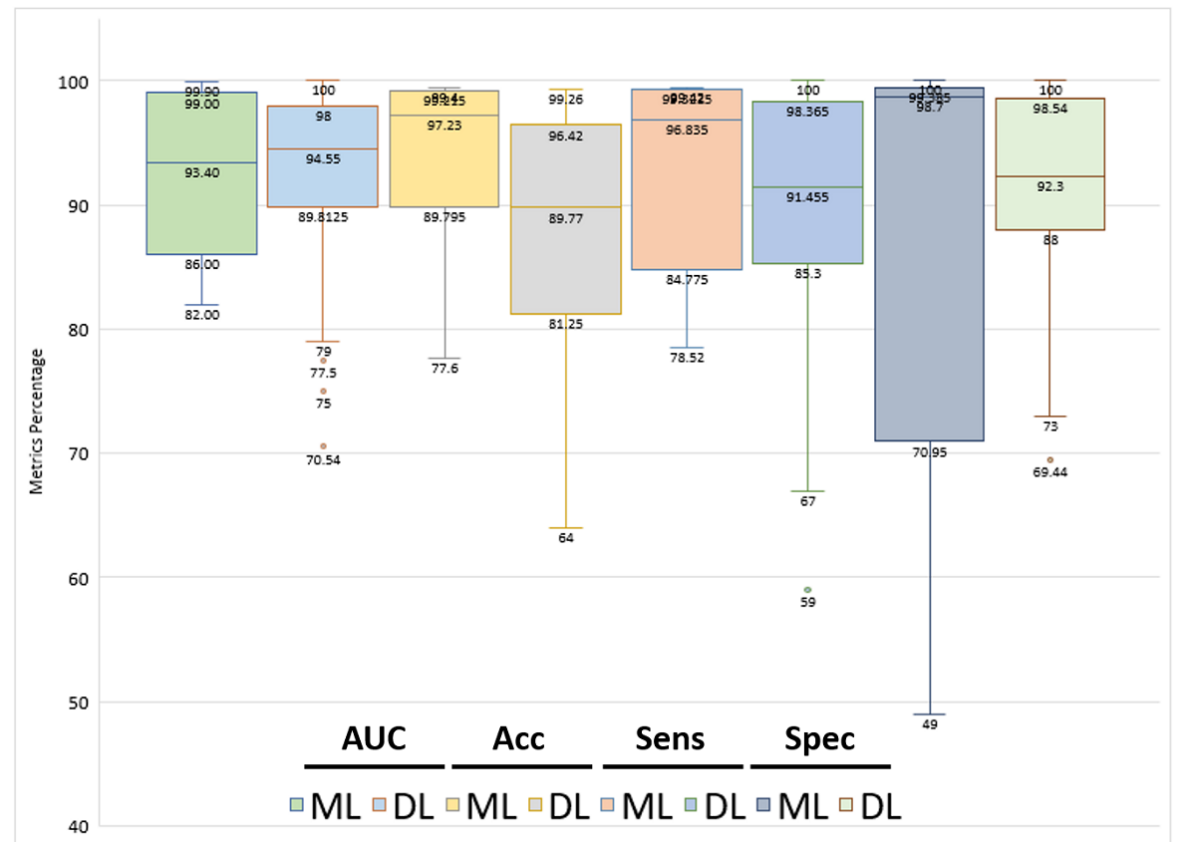
Individual performances of machine learning (ML)–based and deep learning (DL)–based methods reviewed. Acc: accuracy; AUC: area under the curve; Sens: sensitivity; Spec: specificity.

We separately analyzed the performance results of ML and DL approaches. Among 11 (7+4) [13,14,37,38,40,43,63,64,67,68,73] studies that used ML, the accuracy ranged from 77.6% [38] to 99.4% [14] with a mean score of 93.71% and median of 97.23%, the AUC ranged from 82% [43] to 99.9% [73] with a mean score of 92.03% and median of 93.4%, the sensitivity ranged from 78.52% [73] to 99.42% [68] with a mean score of 92.55% and median of 96.835%, and the specificity ranged from 49% [40] to 100% [73] with a mean score of 87.01% and median of 98.7%. Meanwhile, among 38 (34+4) studies that used DL, the accuracy ranged from 64% [45] to 99.26% [68] with a mean score of 87.83% and median of 89.77%, the AUC ranged from 70.54% [20] to 100% [58] with a mean score of 92.12% and median of 94.55%, the sensitivity ranged from 59% [51] to 100% [58] with mean score of 89.84% and median of 91.455%, and the specificity ranged from 69.44% [44] to 100% [17,58,73] with a mean score of 91.54% and median of 92.3%. Figure 7 shows the individual performance results for each ML and DL approach.

**Figure 8:**
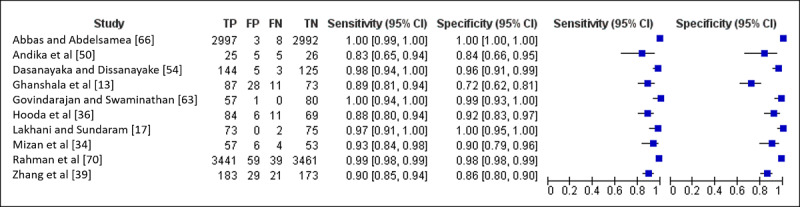
Forest plot of pooled sensitivity and specificity of the 10 included studies. FN: false negative; FP: false positive; TN: true negative; TP: true positive.

In the meta-analysis phase, we analyzed 10 of the included studies [13,17,34,36,39,50,54,63,66,70] that provided confusion matrix results. Among those 10 included studies, 2 used ML approaches, such as SVM, RF, kNN [13], extreme learning machine (ELM), and online sequential ELM [63], while 8 [17,34,36,39,50,54,66,70] used various DL methods, particularly CNN architectures, such as AlexNet, GoogLeNet, VGG16, DenseNet, ResNet, and MobileNet to name a few. A total of 14,521 observations were classified including 7148 true positives, 142 false positives, 104 false negatives, and 7127 true negatives. Review Manager (RevMan; The Nordic Cochrane Centre, The Cochrane Collaboration) software [78] was utilized to conduct the analysis and create the forest plot as shown in Figure 8. All 10 studies revealed high sensitivity and moderate to high specificity. To conclude, the pooled estimate of sensitivity is 0.9857 (95% CI 0.9477-1.00) and the pooled estimate of specificity is 0.9805 (95% CI 0.9255-1.00).

## Discussion

### Principal Findings

In this SLR, we reviewed available evidence related to the usage and performance of both ML and DL methods for TB detection, particularly on CXR images. Most included studies have recently been published, and only 2 studies [37,43] were published before 2016. Around 2785 Google Scholar citations were recorded for all the included studies by March 21, 2022, where the top 3 cited publications were Lakhani and Sundaram [17] with 1131 citations, van Ginneken et al [43] with 289 citations, and Pasa et al [59] with 175 citations. This confirms the increasing popularity of ML and DL implementation in the medical field, especially for TB disease detection using CXR.

Various CXR data sets have been used in the included studies. Three of the most popular ones are SZ (n=36), MC (n=29), and ChestX-ray14 (n=4). Particularly, SZ and MC data sets are the most widely used as they are available to the public in Jaeger et al’s [32] publication. There are 662 (326 normal and 336 TB) CXR images in SZ, while for MC there are 138 (80 normal and 58 TB) CXR images. Hence, they are considered as small data sets because the total number of both data sets is less than 1000 (800 to be precise). By contrast, ChestX-ray14 contains around 112,120 CXR images of 30,805 patients [79], which is considered as a large data set [80-82]. However, it serves common thorax diseases [33] and is commonly used to add data for classes other than TB. Therefore, application to TB disease detection requires proper data curation [69].

In terms of the performance results, ML showed higher accuracy (mean ~93.71%) and sensitivity (~92.55%), while on average DL models achieved better AUC (~92.12%) and specificity (~91.54%). The ML methods tend to have better accuracy because the feature engineering phase is usually validated by experts. Moreover, they are carefully tuned with different parameters settings. By contrast, both feature engineering and parameter tuning in the DL are automatically done by the deep networks’ architecture without human intervention [83]. However, the DL approach has better AUC than the ML approach. This metric is commonly used in medical settings to evaluate the predictive performance of a classifier [84]. It is considered a better evaluation metric than accuracy [84,85], especially when used in imbalanced data settings [86].

From the grouped box plots shown in Figure 7, both ML and DL seem to have similar performance results in general. However, 2 performance metrics, namely, accuracy and specificity, have different interquartile ranges. This suggests that the developed ML and DL methods in the included studies have different level of agreement in terms of reported accuracy and specificity. The accuracy of studies that applied ML has a better level of agreement than DL but the box plot is negatively skewed. It means that among the included studies that applied ML, more studies reported lower accuracy results with wider variance than those that reported higher accuracy results above the median value. The similar finding for specificity can be deducted from the box plot of ML which is very negatively skewed than DL. In general, DL has a better level of agreement for most performance metrics results of included studies as indicated by the shorter interquartile ranges than ML with the exception for accuracy. Therefore, DL tends to give a more stable and consistent result than ML for TB detection.

Both ML and DL have high sensitivity (ML ~92.55%/DL ~89.84%) and specificity (~87.01%/~91.54%). Further analysis on the 10 included studies that provided confusion matrix results confirms this finding. The pooled sensitivity is 0.9857 and the pooled specificity is 0.9805, which once again shows the potential value of ML and DL approaches for TB detection using CXR. A more complete data summary is provided in Multimedia Appendix 3.

Another important factor that might influence the performance results of the DL approach is the data volume used in the learning phase. It is a well-known fact that a lot of data number is needed in the training phase for a DL method to work well [87,88]. However, as previously stated, most of the included studies used the SZ and MC data sets, which are considered small data sets. One possible immediate solution is to apply data augmentation techniques to increase the data volume. Data augmentation is regarded as a data-space solution that could enhance both data size and quality to increase DL performance [89]. Some popular data augmentation techniques are kernel filters, geometric transformations, random erasing, mixing images, color and feature space transformations, and even DL-based data augmentation, such as the variational autoencoders [90,91] and generative adversarial network [89,92].

Transfer learning is another approach that can be utilized to handle insufficient data volume. It transfers the knowledge from source to target domain by relaxing the hypothesis that training data should be independent and identically distributed with test data [93,94]. Using this approach, the dependency on target domain large data volume can be reduced [95]. Unarguably, this approach has been largely applied in many included studies in this SLR, as can be seen in the utilization of various pretrained DL models with promising results, such as Mizan et al [34], Hwang et al [35], Abbas and Abdelsamea [66], Kim et al [69], and Rahman et al [70].

Another interesting finding from this SLR is that the use of multiple input data types (multimodal) could enhance the performance results of both ML and DL than using only 1 input type (unimodal, ie, CXR alone). The other input data types are clinical features [40], demographic information [19], and even other images, such as microbiological and computed tomography [96,97]. This is in line with the conclusion of several other studies [98-100]. Particularly, 6 out of the 47 included studies in this SLR used the multimodal approach than the unimodal approach. Melendez et al [40] used CXR and 12 other clinical features, such as BMI, axillary temperature, heart rate, mid-upper arm circumference, HIV status, anemic conjunctivae, lung auscultation findings, cough, hemoptysis, night sweats, dyspnea, and chest pain. Ahsan et al [41] and Owais et al [48] used CXR images together with their text attributes (age, gender, and TB state). Similarly, Rajpurkar et al [46] used CXR and 8 clinical features (age, oxygen saturation, hemoglobin, CD4 T-cell count, white blood cell count, temperature, current antiretroviral therapy status, and patients’ previous history of TB). Gozes and Greenspan [52] utilized both CXRs and their metadata (age, gender, and patients’ position), while Heo et al [19] used CXR and demographic information (age, gender, weight, and height).

It is worth noting that most of the included studies focused on the development of ML and DL models or architectures as the proposed solution. Only 2 studies [46,55] have developed and built an application or running prototype as the proposed solution in detecting TB disease based on CXR. Although there is some commercial DL software available, they are mainly utilized in high-resource environments. Hence, further development and implementation of a running application are needed, especially in low-resource settings.

The absence of longitudinal (temporal) aspect of the data sets could not be ignored in this study. In practice, the diagnostic decision of TB detection by a medical practitioner or a radiologist using CXR is generally made by detecting change in a lesion compared with the previous observation. However, none of the included studies have considered the longitudinal dimension of the data sets used when building the model and making the decision. Hence, the proposed approaches are prone to false prediction, especially when used on an older age group and people with a history of TB.

We also assessed the publication bias and applicability of the 47 included studies using the QUADAS-2 tool. Among 4 key aspects in the Risk of Bias assessment, namely, patient selection, index test, reference standard, and flow and timing, most included studies had low risks. However, it is important to note that 17 studies were regarded as having unclear risks for the reference standard aspect, and 6 studies as having unclear risks for the flow and timing aspect. This is mainly because no explicit and clear information was given about reference standards, time intervals, and any intervention being conducted in those studies. Hence, future studies should pay close attention to these 2 aspects of risk of bias because they could affect our confidence with the studies’ results.

Particularly, the missing information about the reference standards’ procedure in determining the pulmonary TB (PTB) disease could really affect the confidence of the performance results achieved by various ML and DL methods. It is generally agreed that the mycobacterial culture is closest to a gold standard while the radiology, even under ideal circumstances, is an imperfect diagnostic tool. Therefore, a human radiologist’s diagnosis, even a consensus diagnosis, is an imperfect criterion. Comparing machine reading with this reference standard may tend to overestimate the “true” accuracy. By contrast, it is conceivable that machine reading might be better than human radiologists at detecting microbiologically confirmed PTB, if the algorithm is trained against microbiologically confirmed cases and controls. The use of a radiological reference standard would fail to detect this benefit.

For the applicability concerns, 3 aspects were assessed for all 47 included studies. We conclude that all the included studies have low concerns regarding applicability. This means that the proposed solutions in the included studies are logically sound, feasible, and applicable in solving the task to detect TB based on CXR using ML and DL approaches.

### Limitations

There are some limitations to this SLR. First, we only utilized 3 major databases in identifying and collecting all publications, namely, Scopus, PubMed, and IEEE. We argue that these databases adequately represent the domain of TB detection using ML and DL approaches on CXR. Next, we did not seek further information on reference standards used in each included study. As most included studies used publicly available CXR data sources, reference standards used in those studies were assumed to be the same. Lastly, we did not include any studies that focused on the use of various AI-powered technologies, such as CAD4TB [101-106], Lunit [107], and qXR [108], without proper explanation about the AI-based algorithms used. This review study was specifically designed around the use of ML and DL for TB detection on CXR. Without enough information on the underlying algorithms used in those AI-powered technologies, insightful investigation related to the review question could not be achieved.

### Conclusions

These findings confirm the high potential of both ML and DL for TB detection using CXR. DL approaches, particularly the CNNs, dominantly applied than ML approaches. Besides, DL approaches tend to have more stable and consistent performance results than ML approaches. Data volume and quality were the main concerns of this review, where most of the included studies used relatively small data sets. Therefore, proper data curation and augmentation, transfer learning, and multimodal approaches could be considered in future studies.

## Appendix

Multimedia Appendix 1 List of Missing Records.

Multimedia Appendix 2 Full Characteristics of Included Studies.

Multimedia Appendix 3 Complete Data Summary.

## Abbreviations

AI: artificial intelligence
AUC: area under the curve
CNN: convolutional neural network
CXR: chest x-ray
DL: deep learning
ELM: extreme learning machine
IEEE: Institute of Electrical and Electronics Engineers
kNN: k-nearest neighbor
LR: logistic regression
MC: Montgomery County
MIL: multiple-instance learning
miSVM: multiple instance support vector machine/ maximum pattern margin support vector machine
ML: machine learning
PRISMA: Preferred Reporting Items for Systematic Reviews and Meta-Analyses
PROSPERO: Prospective Register of Systematic Reviews
PTB: pulmonary tuberculosis
QUADAS-2: Quality Assessment of Diagnostic Accuracy Studies version 2
RF: random forest
SLR: systematic literature review
SVM: support vector machine
SZ: Shenzhen

## Data Availability

All data generated or analyzed during this study are included in this published article and its supplementary information files.
