# Supplementary material for: Machine and Deep Learning for Tuberculosis Detection on Chest X-Rays: Systematic Literature Review
Source: J Med Internet Res. 2023 Jul 3;25:e43154. doi: 10.2196/43154 (PMC10365622; doi:10.2196/43154)
Supplement: Multimedia Appendix 1 [file jmir_v25i1e43154_app1.doc]

**Scopus**:

1. Role of chest radiograph (Cxr) in covid-19 diagnosis and management
2. Clinical study of pulmonary tuberculosis in diabetes mellitus
3. Evaluation of hormonal changes in menstrual cycle of women infected with pulmonary tuberculosis in Nnewi, south eastern Nigeria
4. Conjunctival Kaposi's sarcoma in HIV-positive heterosexual Nigerian woman - A case report
5. Adenosine Deaminase in the Diagnosis of Tuberculous Pericardial Effusion
6. Diagnosis and treatment of latent tuberculosis infection
7. Felty's syndrome with pulmonary tuberculosis
8. Tuberculous peritonitis: Analysis of 17 cases
9. The clinical and laboratory profile of tuberculous empyema
10. Disseminated histoplasmosis in a patient with acquired immunodeficiency syndrome (AIDS): A case report
11. Pulmonary function tests and airway responsiveness to methacholine in chronic bronchiectasis of the adult

**PubMed**

1. Diabetes among new cases of pulmonary tuberculosis in Hanoi, Vietnam
2. Usefulness of Quantiferon-TB Gold in Tube® in screening for latent tuberculosis infection in health workers
3. Disseminated histoplasmosis in a patient with acquired immunodeficiency syndrome (AIDS): a case report
4. Adenosine Deaminase in the Diagnosis of Tuberculous Pericardial Effusion
5. A study on low performance status cases of pulmonary tuberculosis in the elderly
6. A case of chronic tuberculous pyothorax associated malignant lymphoma
7. A case of MPO ANCA associated glomerulonephritis with interstitial pneumonitis complicated with lung tuberculosis and pericarditis
8. A case of pulmonary, pleural, and renal tuberculosis associated with DIC and a prolonged increase in D-dimer
