# Supplementary material for: Machine and Deep Learning for Tuberculosis Detection on Chest X-Rays: Systematic Literature Review
Source: J Med Internet Res. 2023 Jul 3;25:e43154. doi: 10.2196/43154 (PMC10365622; doi:10.2196/43154)
Supplement: Multimedia Appendix 2 [file jmir_v25i1e43154_app2.docx]

**Table S1**. Full characteristics of included studies.

| **No** | **Authors** | **Objectives** | **Findings** | **Dataset** | **ML-DL** | **Performance Metrics** |
| --- | --- | --- | --- | --- | --- | --- |
| 1 | Mizan et al (2020) [34] | To detect Tuberculosis using four pretrained CNN models and compare their performances. | DenseNet-169 performed best among the four architectures and got 91.6% validation accuracy, 92% precision, 92% recall, 92% F1-score and AUC score of 0.915. | SZ, MC | CNNs: DenseNet-169, MobileNet, Xception, Inception-V3 | Precision, Recall, F1-Score, Validation Accuracy, AUC |
| 2 | Hwang et al (2016) [35] | To design a CAD system based on deep CNN for automatic TB screening. | Experimental results show that the proposed system has high screening performance in terms of various performance metrics. | KIT, MC, SZ | Customized CNN based on AlexNet + Transfer Learning | Accuracy, AUC, Average Precision |
| 3 | Hooda et al (2020) [36] | To present a deep ensemble architecture-based CAD system to perform PTB classification. | The performance achieved by the ensemble is significantly higher than each of the individual architecture. | MC, SZ, Belarus, JSRT | Proposed (Blocks), AlexNet, ResNet, Ensemble (Proposed+AlexNet+ResNet) | Accuracy, Sensitivity, Specificity, AUC |
| 4 | Melendez et al (2015) [37] | To investigate an alternative machine learning approach, known as multiple-instance learning (MIL), that does not require labeling of each feature sample during training but only a global class label characterizing a group of samples and to propose an improved algorithm that overcomes miSVM's drawbacks related to positive instance underestimation and costly iteration. | After retraining, the MIL-based system significantly outperformed the supervised one. | Zambia, Tanzania, Gambia | kNN, MIL-based system: miSVM, miSVM+PEDD, si-miSVM+PEDD | AUC |
| 5 | Rajaraman et al (2018) [38] | To evaluate the performance of a stacked ensemble that optimally combined classifiers using hand engineered features with those extracted from pre-trained CNNs through two different proposals for improving the accuracy of TB detection in PA CXR images. | The stacked ensemble of models using local and global feature descriptors and pre-trained CNNs could be a promising option for improving the detection accuracy. | SZ, MC, Kenya, India | 1) SVM with GIST, HOG, SURF (Feature Engineering), 2) SVM with AlexNet, VGG-16, GoogleNet, ResNet-50, 3) Ensemble approach | Accuracy, AUC |
| 6 | Zhang et al (2020) [39] | To focus on the task of locating or pinpointing TB by introducing the attention mechanism into CNN networks. | The proposed method performance is better than the models used in previous work. | Jilin, Guangzhou, Shanghai | Proposed: Feed-forward CNN model with integrated CBAM, 4 other CNNs (AlexNet, GoogleNet, DenseNet, ResNet-50) | Accuracy, Sensitivity, Specificity, AUC |
| 7 | Melendez et al (2016) [40] | To propose a novel strategy to combine automatic CXR scoring by CAD with clinical information, including symptoms and HIV status. | Combining CAD and clinical information to estimate the risk of active disease is a promising tool for TB screening. | Cape Town | Feature engineering: mRMR - multiple learner fusion: RF and ERT | AUC, Sensitivity, Specificity, NPV |
| 8 | Ghanshala et al (2020) [13] | To propose image analysis-based framework using various machine learning techniques like SVM, kNN, Random Forest and Neural Network for effective identification of tuberculosis. | The proposed framework using neural network was able to classify better than other classifiers to detect Tuberculosis and achieves accuracy of 80.45%. | MC, SZ, JSRT | SVM, RF, kNN, Neural Network | AUC, F1-Score, Precision, Recall, CA (classification accuracy?) |
| 9 | Ahsan et al (2019) [41] | To develop a generalized model which takes care of all the complex preprocessing steps done by a conventional decision tree approach. | VGG-16 can use the raw data to classify the results with comparable accuracy without any form of pre-processing. Moreover, accuracy increases when VGG-16 is applied on augmented images. | MC, SZ | CNN: VGG-16 | Accuracy, AUC |
| 10 | Sharma et al (2020) [42] | To create efficient deep learning models, trained with chest X-ray images, for rapid screening of COVID-19 and other infectious diseases including TB. | AI-based classification models trained through the transfer learning approach can efficiently classify the CXR images representing studied diseases (normal, COVID-19, non-COVID-19, pneumonia, and tuberculosis images). | Custom dataset | 29 AI deep models (27 plus, dataset 27 trained with two additional epoch sizes) | Accuracy |
| 11 | Hooda et al (2019) [18] | To present an ensemble deep-learning-based TB detection system which achieves significantly high accuracy. | The ensemble attains the accuracy of 88.24% and area under the curve is equal to 0.93, which eclipses the performance of most of the existing methods. | MC, SZ, Belarus, JSRT | Ensemble of AlexNet, GoogleNet, ResNet | Accuracy, Sensitivity, Specificity, AUC ROC |
| 12 | van Ginneken et al (2002) [43] | To detect abnormalities in frontal chest radiographs which are aggregated into an overall abnormality score. | Although not all classes of abnormal findings from this TB screening were used in the experiments, the results suggest that this method may be helpful to radiologists for reading mass chest screening images. | Netherlands, ID database | ASM segmentation, kNN classifier, weighted multiplier | Sensitivity, Specificity, AUC ROC |
| 13 | Chandra et al (2020) [14] | To propose an automatic technique for detection of abnormal CXR images containing one or more pathologies like pleural effusion, infiltration, fibrosis, hila enlargement, dense consolidation, etc. due to tuberculosis. | Results show the promising performance of the proposed technique for TB detection compared to the existing state of the art approaches. Further, the obtained results are statistically validated using Friedman post-hoc multiple comparison methods, which confirms the significance of the proposed method. | MC, SZ | SVM with hierarchical feature extraction | Accuracy, Precision, Recall, F1, MCC, AUC, Error Rate |
| 14 | Karnkawinpong and Limpiyakorn (2018) [44] | To use CNNs, a type of deep learning that employs multiple hidden layers and has been remarkably successful for image classification, to detect whether having a TB lesion or not on the CXR images. | The result showed that CapsNet outperformed the other models when predicting affined images. | MC, SZ, Thailand | AlexNet, VGG-16, CapsNet | Accuracy, Sensitivity, Specificity |
| 15 | Stirenko et al (2018) [45] | To describe the proposed lung segmentation technique in combination with lossless and lossy data augmentation which allow us to get the statistically reliable predictions of lung diseases (availability of tuberculosis) for such a small dataset (<10000 images) even. | Besides the more complex deep CNNs and bigger datasets, the better progress of CADx for the small and not well-balanced datasets even could be obtained by better segmentation, data augmentation, dataset stratification, and exclusion of non-evident outliers. | SZ | Customized CNN | Accuracy |
| 16 | Rajpurkar et al (2020) [46] | To develop a TB diagnostic algorithm and assistant to help clinicians in the diagnosis of TB using chest x-rays in patients co-infected with HIV. | Deep learning assistance may improve clinician accuracy in TB diagnosis using chest x-rays, which would be valuable in settings with a high burden of HIV/TB co-infection. Moreover, the high accuracy of the stand-alone algorithm suggests a potential value particularly in settings with a scarcity of radiological expertise. | Africa | Customized CNN based on DenseNet-121 | Accuracy, Sensitivity, Specificity |
| 17 | Sivaramakrishna et al (2018) [47] | To evaluate the performance of one customized model and five pre-trained CNNs towards improving the accuracy of TB screening using frontal CXRs. | Pretrained CNNs are a promising feature extracting tool for medical imagery including the automated diagnosis of TB from chest radiographs but emphasize the importance of large data sets for the most accurate classification. | SZ, MC, Kenya, India | Customized CNN, AlexNet, VGG-16, VGG-19, Xception, ResNet-50 | Accuracy, AUC |
| 18 | Owais et al (2020) [48] | To propose a comprehensive CAD framework for the effective diagnosis of TB by providing visual as well as descriptive information from the previous patients’ database. | The classification model exhibited the best diagnostic performance and outperforms the performance of various state-of-the-art methods. | SZ, MC | Ensemble-shallow–deep CNN (ensemble-SDCNN) + MLSM algorithm | Accuracy, AP, AR, F1 Score, AUC |
| 19 | Xie et al (2020) [49] | To propose the first multicategory tuberculosis lesion detection method. | The proposed computer-aided system is superior to current systems that can be used to assist radiologists in diagnoses and public health providers in screening for tuberculosis in areas where tuberculosis is endemic. | JSRT, SZ, MC, local from the FAHXJU | Segmentation: U-Net, Classification: Proposed method based on Faster RCNN + feature pyramid network (FPN) | AUC, Accuracy, Sensitivity, Specificity |
| 20 | Andika et al (2019) [50] | To use deep learning CNN method with Adam optimizer to detect PTB. | The best model to classify PTB disease using CNN is built using 75 epochs which accuracy results in the training and validation data are 99.19% and 80.60% respectively. The model then implemented into test data with an accuracy rate of 84%. | SZ | Customized CNN | Precision, Recall, Accuracy |
| 21 | Das et al (2020) [51] | To showcase a cross-population validation test on a deep learning model and to validate its robustness across data from two different parts of the world. | Unlike feature engineering, the method is flexible enough to adjust and improve overall performance of the Tuberculosis screening in decision-making process. | SZ, MC | InceptionNet V3 and modified (truncated) InceptionNet V3 | Accuracy, Sensitivity, Specificity, Precision, AUC |
| 22 | Gozes and Greenspan (2019) [52] | To present a feature learning scheme which uses pathology labels and metadata (gender, age, patient's position) of a hospital-scale chest X-ray dataset. | In the experiments on small-scale datasets, the authors have demonstrated the advantage of the proposed architecture (MetaChexNet) over ImageNet pre-trained architectures in both detection results and generalization ability on an external dataset. | ChestXray14, MC, SZ | MetaChexNet based on DenseNet-121 | AUC |
| 23 | Hooda et al (2017) [53] | To present a potential method for tuberculosis detection using deep-learning which classifies CXR images into two categories, that is, normal and abnormal. | Adam optimizer with an overall accuracy of 94.73% and validation accuracy of 82.09% performed best amongst three optimizers experimented. | SZ, MC | Proposed CNN | Accuracy, Cross-entropy loss |
| 24 | Heo et al (2019) [19] | To use deep learning to detect tuberculosis in chest radiographs in annual workers’ health examination data and compare the performances of CNNs based on images only (I-CNN) and CNNs including demographic variables (D-CNN). | The results indicate that machine learning can facilitate the detection of tuberculosis in chest X-rays, and demographic factors can improve this process. | Yonsei | VGG19, InceptionV3, ResNet50, DenseNet121, InceptionResNetV2 and D-CNN (VGG19 + demographic variables) | AUC |
| 25 | Lakhani and Sundaram (2017) [17] | To evaluate the efficacy of deep convolutional neural networks (DCNNs) for detecting tuberculosis (TB) on chest radiographs. | Deep learning with DCNNs can accurately classify TB at chest radiography with an AUC of 0.99. | SZ, MC, Belarus, Thomas Jefferson University Hospital | Ensemble of AlexNet and GoogleNet | Accuracy, Sensitivity, Specificity, AUC ROC |
| 26 | Sathitratanacheewin et al (2020) [20] | To develop a Deep Convolutional Neural Network (DCNN) model using a Tuberculosis (TB)-specific chest x-ray (CXR) dataset of one population (National Library of Medicine Shenzhen No.3 Hospital) and tested it with non-TB-specific CXR dataset of another population (National Institute of Health Clinical Centers). | A supervised deep learning model developed by using the training dataset from one population may not have the same diagnostic performance in another population. Technical specification of CXR images, disease severity distribution, dataset distribution shift, and overdiagnosis should be examined before implementation in other settings. | SZ, ChestX-ray8 | Proposed CNN based on Inception V3 | AUC |
| 27 | Dasanayaka and Dissanayake (2020) [54] | To present a highly accurate, automated TB screening system using chest X-rays, which would be helpful especially for low income countries with low access to qualified medical professionals. | The automated TB detection model presented in this work is reliable. | SZ, MC, MIMIC, and Synthesis | Proposed CNN based on GAN, UNET and Ensemble of (VGG16 + InceptionV3) | Accuracy, Sensitivity, Specificity, Youden's index |
| 28 | Nguyen et al (2019) [55] | To propose an improved method for transfer learning and a new method for obtaining low level features by training the models in a multiclass multilabel scenario. | ImageNet weights are insufficient and the usage of appropriate data for pre-training is important and makes the entire process more efficient. | SZ, MC, NIH-14 | ResNet-50, VGG16, VGG19, DenseNet-121, Inception ResNet | AUC |
| 29 | Meraj et al (2019) [56] | To implement four CNNs in identification of TB manifest CXRs. | Proposed VGG-16 model has gained highest score overall compared to the models from other two previous studies. | SZ, MC | VGG-16, VGG-19, ResNet50, GoogleNet | Accuracy, AUC |
| 30 | Becker et al (2018) [57] | To evaluate the feasibility of Deep Learning- based detection and classification of pathological patterns in a set of digital photographs of CXR images of TB patients. | Deep Learning analysis of CXR photographs is a promising tool. Further efforts are needed to build larger, high-quality data sets to achieve better diagnostic performance. | Uganda | ViDi - industrial-grade Deep Learning image analysis software (Suite v2.0, ViDi Systems, Villaz-Saint-Pierre, Switzerland) | AUC, Sensitivity, Specificity, PPV |
| 31 | Hwang et al (2018) [58] | To develop a deep learning–based automatic detection algorithm (DLAD) for active pulmonary tuberculosis on CRs and to validate its performance using various datasets in comparison with that of physicians. | The DLAD demonstrated excellent and consistent performance in the detection of active pulmonary tuberculosis on CR, outperforming physicians, including thoracic radiologists. | SNUH, Boramae, Kyunghee, Daejeon Eulji, MC, SZ | Proposed CNN | AUC, AUAFROC, True detection rate, Sensitivity, Specificity |
| 32 | Pasa et al (2019) [59] | To propose a simple convolutional neural network optimized for TB diagnosis which is faster and more efficient than previous models but preserves their accuracy. | The specialized architecture achieve good results compared to other publications, while reducing the computational, memory and power requirements significantly. The saliency maps offer a good visual explanation of the network decision. | MC, SZ, Belarus | Proposed CNN | Accuracy, AUC |
| 33 | Ahmad Hijazi et al (2020) [60] | To present an approach to ensemble different architectures of deep learning for TB detection. | The ensemble of the three classifiers using majority voting, produced the best TB detection performance. | SZ, MC | Ensemble of InceptionV3, VGG-16, and a custom-built architecture | Accuracy, Sensitivity, Specificity |
| 34 | Hwa et al (2019) [61] | To present an ensemble deep learning for TB detection using chest x-ray and Canny edge detected images. | Using different types of features extracted from different types of images can improve the detection rate. | SZ, MC | Ensemble of InceptionV3, VGG-16 | Accuracy, Sensitivity, Specificity |
| 35 | Ayaz et al (2021) [62] | To propose a novel TB detection technique that combines hand-crafted features with deep features (convolutional neural network-based) through Ensemble Learning. | The proposed methodology achieved significant improvement in results. The Ensemble worked better than individual classifiers. | SZ, MC | Ensemble (pretrained CNNs: InceptionV3, InceptionResnetv2, VGG16, VGG19, MobileNet, ResNet50, Xception) with Gabor filter | Accuracy, AUC |
| 36 | Govindarajan and Swaminathan (2021) [63] | To identify and classify Tuberculosis conditions from healthy subjects in chest radiographs using integrated local feature descriptors and variants of extreme learning machine. | ELM based method is able to differentiate the subtle changes in inter and intra subject variations of CXR images, the proposed methodology seems to be useful for computer-based detection of PTB. | MC | Extreme Learning Machine (ELM), Online Sequential ELM (OSELM) | Accuracy, Sensitivity, Specificity, Precision, F-score, MCC |
| 37 | Rashid et al (2018) [64] | To present RID network that could distinguish between normal and TB infected radiograph. | The algorithm achieved 90.5% average accuracy that is among top accuracies achieved and hence, proved its robustness and competence. | SZ | Ensemble of ResNet-152, Inception-ResNet-v2, DenseNet-161 + SVM | Accuracy, Sensitivity, Specificity, AUC ROC |
| 38 | Munadi et al (2020) [65] | To evaluate the effect of two different pre-processing approaches (UM and HEF) on the use of pre-trained CNN to detect TB disease. | The use of an image enhancement system to pre-process the TB images will thus allow the tested pre-trained network to learn better model. | SZ | Image enhancements: UM, HEF, CLAHE - DL: ResNet-50, EfficientNet-B4, ResNet-18 | Accuracy, AUC |
| 39 | Abbas and Abdelsamea (2018) [66] | To study the effect of knowledge transformation from ImageNet using a pre-trained model such as AlexNet on CXR images for the purpose of identification of manifestation of TB. | Using fine-tuning technique outperformed both shallow-tuning and deep-tuning techniques and achieved 0.998 for the AUC, 0.999 for specificity, and 0.997 for sensitivity rate. | MC | AlexNet | Sensitivity, Specificity, AUC ROC |
| 40 | Melendez et al (2016) [67] | To propose an improved algorithm for training a MIL classifier that builds upon concepts from other machine learning paradigms, such as AL and one-class classification, to achieve a more accurate low-level output. | The detection performance of a MIL-based CAD system, as measured through pixel classification, has been substantially improved. | Zambia | MIL + AL | AUC |
| 41 | Khatibi et al (2021) [68] | To propose a novel multi-instance classification model which is based on CNNs, complex networks and stacked ensemble (CCNSE) for tuberculosis recognition. | The proposed method can be used as a computer-aided diagnosis system to reduce the manual time, effort and dependency to specialist’s expertise level. | MC, SZ | LR, SVM with linear (SVM-L) and radial basis function (SVM-RBF) kernels, DT, RF and Adaboost - CNNs: VGG-16, VGG-19, ResNet-101, ResNet-150, DenseNet, Xception | Accuracy, AUC, Sensitivity, Specificity |
| 42 | Kim et al (2019) [69] | To determine whether unlabeled datasets can be used to further train and improve the accuracy of a deep learning system (DLS) for the detection of tuberculosis (TB) on chest radiographs (CXRs) using a two-stage semi-supervised approach. | Using semi-supervised learning, the authors trained a deep learning algorithm that detected TB at a high accuracy and demonstrated value as a CAD tool by identifying relevant CXR findings, especially in cases that were misinterpreted by radiologists. | ChestXray14, MC, SZ, JHH | ResNet-50, TBNet | Sensitivity, Specificity, PPV, NPV, AUC |
| 43 | Rahman et al (2020) [70] | To focus on the detection of TB using transfer learning-based technique of CNNs on the original and segmented lungs in X-ray images. | The proposed method with state-of-the-art performance can be useful in the computer-aided faster diagnosis of tuberculosis | Kaggle, NLM, Belarus, NIAID TB dataset, RSNA CXR dataset | Lung segmentation - U-Net, classification - MobileNetv2, SqueezeNet, ResNet18, Inceptionv3, ResNet 50, ResNet101, CheXNet, VGG19, and DenseNet201 | Accuracy, Precision, Sensitivity, F1-score, Specificity |
| 44 | Yoo et al (2020) [71] | To suggest two-step of process for detection of normal, TB and non-TB disease using binary classifier for each step. | Re-training could improve the stability of prediction accuracy for images in different data groups. | ChestX-ray14, SZ, East Asian Hospital | ResNet18 | Accuracy, Sensitivity, Specificity, Precision, AUC |
| 45 | Oloko-Oba and Viriri (2020) [72] | To propose a model that employs the use of learning algorithm (CNN) to effectively learn the features associated with tuberculosis and make corresponding accurate predictions. | The model expresses a promising pathway in solving the diagnosis issue in early detection of tuberculosis manifestation and, hope for the radiologists and medical healthcare facilities in the developing countries. | SZ | Proposed ConvNet | Accuracy |
| 46 | Guo et al (2020) [73] | To propose an integrated process to improve TB diagnostics via convolutional neural networks (CNNs) and localization in CXRs via deep-learning models. | Compared to the state-of-the-art, the resulting approach showcases an outstanding performance both in the lung abnormality detection and the specific TB-related manifestation diagnosis vis-à-vis the localization in CXRs. | SZ, NIH | ABC - VGG16, VGG19, Inception V3, ResNet34, ResNet50, and ResNet101 - Proposed ensemble CNN | Accuracy, Specificity, Recall, F1, AUC |
| 47 | Ul Abideen et al (2020) [74] | To present the solution for TB identification by using Bayesian-based convolutional neural network (B-CNN). | Results prove the supremacy of B-CNN for the identification of TB and non-TB sample CXRs as compared to counterparts in terms of accuracy, variance in the predicted probabilities and model uncertainty. | SZ, MC | Proposed B-CNN | Accuracy |

**Table S1**. Full characteristics of included studies (continued).

| **No** | **Authors** | **Best Result** | **Comparison with Other Studies** | **Outcome Type** | **Google Scholar Citation** | **Funding/ Sponsor** |
| --- | --- | --- | --- | --- | --- | --- |
| 1 | Mizan et al (2020) [34] | **DenseNet-169**: Prec 92%, Rec 92%, F1 92%, Val Acc 91.67%, AUC 0.915 | Yes (4 other studies) | Model/ architecture | 0 | N/A |
| 2 | Hwang et al (2016) [35] | **Customized CNN**: AUC 96.7% (SZ), Acc. 90.5% (MC) | N/A | Model/ architecture | 151 | This work was supported in part by The Korean Institute of Tuberculosis (KIT) under Korean National Tuberculosis Association (KNTA), and in part by Korea Digital Hospital Export Agency (KOHEA) |
| 3 | Hooda et al (2020) [36] | **Ensemble**: Acc 90.0%, AUC 0.96, Sens 88.42%, Spec 92.0% | Yes (7 other studies) | Model/ architecture | 0 | N/A |
| 4 | Melendez et al (2015) [37] | **si-miSVM+PEDD**: 0.86 (Zambia), 0.86 (Tanzania), 0.91 (Gambia) | Yes (3 other studies) | Model/ architecture | 91 | N/A |
| 5 | Rajaraman et al (2018) [38] | **Ensemble**: SZ (Acc 93.4%, AUC 0.991), MC (Acc 87.5%, AUC 0.962), K (Acc 77.6%, 0.826), I (Acc 96.0%, AUC 0.965) | Yes (4 other studies) | Model/ architecture | 44 | This research is supported by the Intramural Research Program of the National Institutes of Health (NIH), National Library of Medicine (NLM), and Lister Hill National Center for Biomedical Communications (LHNCBC). |
| 6 | Zhang et al (2020) [39] | **Proposed network**: Recall/Sens 89.7%, Spec 85.9%, Acc 87.7%, AUC 0.943 | N/A | Model/ architecture | 2 | This work is supported by the National Natural Science Foundation of China (No. 81871508; No. 61773246); Taishan Scholar Program of Shandong Province of China (No. TSHW201502038); Major Program of Shandong Province Natural Science Foundation (ZR2019ZD04, No. ZR2018ZB0419). |
| 7 | Melendez et al (2016) [40] | **Multiple learner fusion (RF + ERT)**: AUC 0.84, Sens 95%, Spec 49%, NPV 98% | N/A | Model/ architecture | 91 | This study was supported by the European and Developing Countries Clinical Trials Partnership (EDCTP) grant: the evaluation of multiple novel and emerging technologies for TB diagnosis, in smear-negative and HIV-infected persons, in high burden countries (TB-NEAT project). European and Developing Countries Clinical Trials Partnership (TB-NEAT; IP.2009.32040.009). |
| 8 | Ghanshala et al (2020) [13] | **NN** - AUC 0.894, Acc. 81.1%, F1 81.1%, Prec 81.1%, Recall 81.1% - Average Acc. 80.45% | N/A | Model/ architecture | 1 | N/A |
| 9 | Ahsan et al (2019) [41] | **VGG-16 + data augmentation** - AUC 0.94, Acc 81.25% | N/A | Model/ architecture | 31 | N/A |
| 10 | Sharma et al (2020) [42] | **Custom deep AI model**: 100% N, 100% CoV-19, 66.67% new CoV-19, 100% non-CoV-19, 93.75% Pneu, 80% TB | N/A | Model/ architecture | 40 | This work was financially supported by the Department of Biotechnology (DBT), Government of India, grants BT/BI/04/001/2018 and BT/BI/25/066/2012. AS acknowledges DBT Apex Biotechnology Information Centre at International Centre for Genetic Engineering and Biotechnology (ICGEB, India), for financial assistance. RS received a fellowship from the Council of Scientific and Industrial Research (CSIR), New Delhi, India. |
| 11 | Hooda et al (2019) [18] | **Ensemble** - Acc 88.24%, AUC 0.93, Sens 88.42%, Spec 88% | Yes (9 other studies) | Model/ architecture | 10 | N/A |
| 12 | van Ginneken et al (2002) [43] | **Proposed scheme with kNN**: Sens 86%, Spec 50%, AUC 0.82 | N/A | Model/ architecture | 289 | The work of B. van Ginneken was supported by the Dutch Ministry of Economic Affairs through the IOP Image Processing program, which made it possible to visit the Kurt Rossmann Laboratories at the University of Chicago, during which period most of this study was carried out. |
| 13 | Chandra et al (2020) [14] | **SVM with hierarchical feature extraction**: MC (Acc 95.6%, AUC 0.95), SZ (Acc 99.4%, AUC 0.99) | Yes (7 other studies) | Model/ architecture | 25 | N/A |
| 14 | Karnkawinpong and Limpiyakorn (2018) [44] | **CapsNet** - Acc 80.06%, Sens 92.72%, Spec 69.44% | N/A | Model/ architecture | 6 | N/A |
| 15 | Stirenko et al (2018) [45] | **Customized CNN**: 64% (Lossy data augmentation), 70% (Lossless data augmentation) | N/A | Model/ architecture | 71 | The work was partially supported by Huizhou Science and Technology Bureau and Huizhou University (Huizhou, P.R.China) in the framework of Platform Construction for China-Ukraine Hi-Tech Park Project #2014C050012001 |
| 16 | Rajpurkar et al (2020) [46] | **CheXaid**: Acc 79%, Sens 67%, Spec 87% | N/A | Model/ architecture + web application | 26 | N/A |
| 17 | Sivaramakrishna et al (2018) [47] | **Proposed pretrained CNNs**: Acc 85.5% (SZ), 75.8% (MC), 69.5% (K), 87.6% (I) - AUC 0.926 (SZ), 0.833 (MC), 0.775 (K), 0.956 (I) | Yes (3 other studies) | Model/ architecture | 23 | This work is supported by the Intramural Research Program of the National Institutes of Health, National Library of Medicine, and Lister Hill National Center for Biomedical Communications. |
| 18 | Owais et al (2020) [48] | **Ensemble** on MC: F1 0.929, AP 0.937, AR 0.921, Acc 92.8%, AUC 0.965 | Yes (15 other studies) | Model/ architecture | 3 | This work was supported in part by the Ministry of Science and ICT (MSIT), Korea, under the ITRC (Information Technology Research Center) support program (IITP-2020-2020-0-01789) supervised by the IITP (Institute for Information & Communications Technology Promotion) and in part by the Bio and Medical Technology Development Program of the National Research Foundation of Korea (NRF) funded by the Korean government, the MSIT (NRF-2016M3A9E1915855). |
| 19 | Xie et al (2020) [49] | **Faster RCNN + FPN**: SZ - AUC 0.941, Acc 90.2%, Sens 85.4%, Spec 95.1% - MC - AUC 0.977, Acc 92.6%, Sens 93.1%, Spec 92.3% - Local FAHXJU - AUC 0.993, Acc 97.4%, Sens 98.3%, Spec 96.2% | Yes (6 other studies) | Model/ architecture | 17 | This work was supported by the Xinjiang Uygur Autonomous Region’s Major Science and Technology Project in 2017, “Study on Comprehensive Evaluation and Key Technologies of Tuberculosis Prevention Service System in Southern Xinjiang, Xinjiang”, under grant 2017A03006-1. This work also was supported by the scientific research project of Education Department of Shaanxi Provincial Government (No. 19JK0808). |
| 20 | Andika et al (2019) [50] | **Customized CNN:** Normal: Prec 83%, Rec 83% - PTB: Prec 84%, Rec 84% - Overall acc 84% | N/A | Model/ architecture | 1 | Universitas Sebelas Maret Surakarta for supporting this research through Grant of Improvement of Research Laboratory Capacity 2019. |
| 21 | Das et al (2020) [51] | **Modified InceptionNet V3**: SZ train MC test: Acc 76.05%, AUC 0.84, Sens 63%, Spec 81%, Prec 89% - MC train SZ test: Acc 71.47%, AUC 0.79, Sens 59%, Spec 73%, Prec 84% - Combined: Acc 89.96%, AUC 0.95, Sens 87%, Spec 93%, Prec 92% | N/A | Model/ architecture | 2 | N/A |
| 22 | Gozes and Greenspan (2019) [52] | **MetaChexNet**: SZ AUC 0.965, MC AUC 0.928, Combined AUC 0.937 | Yes (1 other study) | Model/ architecture | 14 | N/A |
| 23 | Hooda et al (2017) [53] | **Proposed CNN**: Acc 82.09%, Loss 0.4013 | Yes (6 other studies) | Model/ architecture | 55 | The authors would like to thank Nvidia Corporation for providing free-of-cost GPU for performing our experiments. We would also like to acknowledge the infra-structural support provided by Design Innovation Center (DIC), UIET, Panjab University, Chandigarh for the execution of this work. |
| 24 | Heo et al (2019) [19] | **D-CNN**: VGG19 AUC 0.9213 - **I-CNN**: VGG19 0.9075 | Yes (1 other study) | Model/ architecture | 55 | This study was supported by a faculty research grant of Yonsei University College of Medicine (6-2018-0175). |
| 25 | Lakhani and Sundaram (2017) [17] | **Ensemble:** AUC 0.99 - **Ensemble + Radiologist augmented**: Sens 97.3%, Spec 100%, Acc 98.7% | Yes (1 other study) | Model/ architecture | 1131 | N/A |
| 26 | Sathitratanacheewin et al (2020) [20] | **Proposed CNN:** SZ AUC 0.8502, ChestX-ray8 AUC 0.7054 | N/A | Model/ architecture | 25 | This study was supported by the Health Systems Research Institute (HSRI 62-103) and Ratchadapiseksompotch Matching Fund, Faculty of Medicine, Chulalongkorn University (RA-MF-12/62). |
| 27 | Dasanayaka and Dissanayake (2020) [54] | **Ensemble:** Youden's index 0.941, Sens 97.9%, Spec 96.2%, Acc 97.1% | N/A | Model/ architecture | 8 | N/A |
| 28 | Nguyen et al (2019) [55] | **DenseNet**: SZ AUC 0.99, MC AUC 0.80 | N/A | Model/ architecture + web application | 23 | NVIDIA Corporation with the donation of the GPUs used for this research. |
| 29 | Meraj et al (2019) [56] | **VGG-16**: - SZ - Acc 86.74%, AUC 0.92 - MC - Acc 77.14%, AUC 0.75 **VGG-19:** AUC 0.90 | Yes (2 other studies) | Model/ architecture | 10 | This work is funded by Universiti Putra Malaysia under Geran Putra (GP/2018/9596100). |
| 30 | Becker et al (2018) [57] | **ViDi software**: Overall AUC 0.98 | N/A | App Testing | 33 | N/A |
| 31 | Hwang et al (2018) [58] | **Proposed CNN:** AUC 0.977 - 1.000, AUAFROC 0.973 - 1.000, Sens 94.3%-100%, Spec 91.1%-100%, True detection rate 94.5% - 100 % | N/A | Model/ architecture | 100 | The work was supported by the Seoul National University Hospital Research fund (grant 04-2016-3000), Lunit Inc., and the Seoul Research & Business Development Program (grant FI170002). |
| 32 | Pasa et al (2019) [59] | **Proposed CNN:** MC - Acc 79.0%, AUC 0.811, SZ - Acc 84.4%, AUC 0.900, Combined 3 datasets - Acc 86.2%, AUC 0.925 | Yes (5 other studies) | Model/ architecture | 175 | This work was supported by the German Research Foundation (DFG) and the Technical University of Munich within the Open Access Publishing Funding Programme. |
| 33 | Ahmad Hijazi et al (2020) [60] | **Ensemble**: Acc 91.0%, Sens 89.6%, Spec 90.7% | N/A | Model/ architecture | 15 | This work was funded by Universiti Malaysia Sabah (UMS) through a grant SDK0018-2017 and Artificial Intelligence Research Unit, UMS. |
| 34 | Hwa et al (2019) [61] | **Ensemble + canny edge:** Acc 89.77%, Sens 90.91%, Spec 88.64% | N/A | Model/ architecture | 15 | Universiti Malaysia Sabah supports this work through a grant SDK0018-2017 and Knowledge Technology Research Unit, UMS. |
| 35 | Ayaz et al (2021) [62] | **Ensemble + gabor filter**: MC Acc 93.47%, AUC 0.97, SZ Acc 97.59%, AUC 0.99 | Yes (9 other studies) | Model/ architecture | 17 | Partial financial support was received by First Author from University of Engineering & Technology Taxila, Pakistan in form of Scholarship and Tuition Fee waiver during this research. |
| 36 | Govindarajan and Swaminathan (2021) [63] | **ELM:** Acc 99.2%, Sens 99.3%, Spec 99.3%, Prec 99.0%, F-score 99.2%, MCC 98.6% - **OSELM:** Acc 98.6%, Sens 98.7%, Spec 98.7%, Prec 97.9%, F-score 98.6%, MCC 97.0% | Yes (6 other studies) | Model/ architecture | 2 | N/A |
| 37 | Rashid et al (2018) [64] | **Ensemble + SVM**: Acc 90.5%, Sens 89.4%, Spec 91.9%, AUC 0.95 | Yes (7 other studies) | Model/ architecture | 8 | N/A |
| 38 | Munadi et al (2020) [65] | **Proposed EfficientNet-B4 + UM**: Acc 89.92%, AUC 0.948 | Yes (4 other studies) | Model/ architecture | 12 | This work was supported in part by the Institute for Research and Community Services (LPPM), Universitas Syiah Kuala, Indonesia, under Grant 9/UN11.2.1/PT.01.03/PNBP/2020, and in part by the Ministry of Research, Technology, and Higher Education of the Republic of Indonesia through the 2019 World Class Professor (WCP) Programme. |
| 39 | Abbas and Abdelsamea (2018) [66] | **AlexNet**: AUC 0.998, Sens 99.7%, Spec 99.9% | Yes (3 other studies) | Model/ architecture | 14 | N/A |
| 40 | Melendez et al (2016) [67] | **MIL + AL**: Pixel-level AUC 0.870 | N/A | Model/ architecture | 38 | N/A |
| 41 | Khatibi et al (2021) [68] | **Proposed SE**: MC Acc 99.26%, AUC 0.99, Sens 99.42%, Spec 99.15% - SZ Acc 99.22%, AUC 0.98, Sens 99.39%, Spec 99.47% | Yes (6 other studies) | Model/ architecture | 4 | N/A |
| 42 | Kim et al (2019) [69] | **TBNet on JHH**: AUC 0.87, Sens 85%, Spec 76%, PPV 0.64, NPV 0.9 - **Majority VoteTBNet and 2 radiologists:** Sens 94%, Spec 85%, PPV 0.76, NPV 0.96 | N/A | Model/ architecture | 8 | This work was supported by Radiological Society of North America R&E Foundation (RMS1816 to TK Kim). |
| 43 | Rahman et al (2020) [70] | W/o segmentation - **ChexNet** Acc 96.47%, Prec 96.62%, Sens 96.47%, F1 96.47%, Spec 96.51% - W segmentation - **DenseNet201** Acc 98.6%, Prec 98.57%, Sens 98.56%, F1 98.56%, Spec 98.54% | Yes (15 other studies) | Model/ architecture | 58 | Open Access funding provided by the Qatar National Library. |
| 44 | Yoo et al (2020) [71] | **ResNet18**: AXIR1 Acc 98%, Sens 99%, Spec 97%, Prec 97%, AUC 0.98 - AXIR2 Acc 80%, Sens 72%, Spec 89%, Prec 87%, AUC 0.80 | N/A | Model/ architecture | 0 | This research was supported by the National Research Foundation of Korea (NRF) grant funded by the Korea government (MSIT) (No. 2020R1A2C4001910 and 2020M2D9A1094075) |
| 45 | Oloko-Oba and Viriri (2020) [72] | **Proposed ConvNet**: Acc 87.8% | Yes (7 other studies) | Model/ architecture | 3 | N/A |
| 46 | Guo et al (2020) [73] | **Ensemble:** SZ Acc 94.59%-98.46%, Spec 95.57%-100%, Rec 93.66%-98.67%, F1 94.7%-98.6%, AUC 0.986-0.999 - NIH Acc 89.56%-95.49%, Spec 96.69%-98.50%, Rec 78.52%-90.91%, F1 85.5%-94.0%, AUC 0.934-0.976 | Yes (6 other studies) | Model/ architecture | 6 | N/A |
| 47 | Ul Abideen et al (2020) [74] | **B-CNN**: MC Acc 96.42%, SZ Acc 86.46% | Yes (7 other studies) | Model/ architecture | 32 | This work was supported by the Higher Education Commission under Grant 2(1064). |
